# Proximity labeling reveals non-catalytic interactions between DPP9 and ubiquitin signaling complexes

**DOI:** 10.1007/s00018-025-06021-z

**Published:** 2026-02-04

**Authors:** Valentina Elena Wirtgen, Layla Saied, Samuel Zolg, Marta Campos Alonso, Bettina Mayer, Laura Donzelli, Ulrich Maurer, H. T. Marc Timmers, Klaus-Peter Knobeloch, Oded Kleifeld, Ruth Geiss-Friedlander

**Affiliations:** 1 Institute of Molecular Medicine and Cell Research, Faculty of Medicine, University of Freiburg, Freiburg, Germany; 2 Faculty of Biology, Technion, Haifa, 32000 Israel; 3 Institute of Neuropathology, Faculty of Medicine, University of Freiburg, Freiburg, Germany; 4 German Cancer Research Center (DKFZ), Department of Urology, German Cancer Consortium (DKTK) partner site Freiburg, Medical Center-University of Freiburg, Freiburg, Germany; 5 Faculty of Biology, University of Freiburg, 79104 Freiburg, Germany

**Keywords:** BRISC complex, CBL, CYLD, DPP8, Proximity labeling, XIAP, MRNA decay, Autophagy

## Abstract

Dipeptidyl peptidase 9 (DPP9) is an amino-peptidase with roles in immunity, DNA-repair, cell signaling, memory and neonatal survival; its dysregulation is linked to cancer and immune-related disorders. While many studies focus on its catalytic activity, scaffolding functions of DPP9 are emerging. Here, we mapped the DPP9 interactome using TurboID-based proximity labeling in DPP9 knock-out HEK293 cells reconstituted with doxycycline-inducible miniTurboID-DPP9, allowing fine-tuned expression that approximates physiological levels. Besides known partners, proteins involved in autophagy, mRNA decay and ubiquitin signaling along with DPP8, were strongly enriched among the identified DPP9 binding partners. Notably, we validated DPP8, the E3 ligase CBL, the deubiquitinase complex CYLD-SPATA2 and the BRISC complex components BRCC36/BRCC3 and ABRO1/ABRAXAS2 as novel DPP9 interactors. Furthermore, NanoBRET assays in living cells demonstrated that DPP9 disrupts the binding between BRCC36/BRCC3 and ABRO1/ABRAXAS2, and the interaction of CYLD with SPATA2, thereby compromising these protein-protein interactions. Mechanistically, these findings reveal physical and potentially regulatory interactions between DPP9 and components of the ubiquitin system and provide a basis for dissecting the non-catalytic functions of DPP9.

**Supplementary Information:**

The online version contains supplementary material available at 10.1007/s00018-025-06021-z.

## Introduction

 Humans express more than 500 distinct proteases that are essential for numerous biological processes. These enzymes, which act predominantly in an irreversible manner, are tightly regulated and their dysregulation is often linked to pathophysiological conditions. Although traditionally the catalytic activity of proteases is studied most, accumulating evidence highlights physiological functions irrespective of their enzymatic activity. These so-called “moonlight functions” are mediated by protein-protein interactions and scaffolding functions rather than substrate cleavage [1]. For example, caspase-8, which is known for its role in apoptosis, also acts as a non-catalytic scaffold that recruits FADD and RIPK1 to form signaling complexes required for NF-κB activation [2]. Similarly, matrix metalloproteinases (MMPs) use their hemopexin and cytoplasmic domains for non-proteolytic signaling [3–6] via interactions with integrins [4], HSP90β [5] or CD44 [6]. Therefore, for a comprehensive understanding of a protease’s function, it is not only critical to identify its substrates but also its broader interactome including regulatory proteins and binding partners whose function is affected in a non-catalytic manner.

Proteases of the S9B dipeptidyl-peptidase 4 (DPP4) family are amino di-peptidases with the rare ability to cleave peptide bonds after proline. This family includes two intracellular homologs, DPP8 and DPP9, which share a similar overall structure [7], and are ubiquitously expressed in various tissues [8, 9]. Of those, DPP9 is more abundant and exists both as a cytoplasmic variant (DPP9-S), and as a nuclear form (DPP9-L) which has a short N-terminal extension with a nuclear localization signal [10–12]. DPP9 is emerging as an attractive drug target due to its observed roles in cell migration [13–15], DNA repair [16], cell signaling [17], mitochondria homeostasis [18, 19], memory [20] and multiple aspects of the immune system [10, 17, 21]. Dysregulation of DPP9 is linked to immunological disorders such as inflammasomopathy and affects dynamics of tumorigenesis and metastasis in cancer [13, 15, 16, 21–27].

Screens for DPP9 substrates have been conducted to better understand its physiological and pathophysiological roles [12, 28]. While several candidates have been identified, only few have been mechanistically characterized. These include antigenic peptides that are processed by DPP9 thereby determining their presentation by MHC class I molecules [10]. Additional substrates are the tyrosine kinase SYK [17], and the tumor suppressor BRCA2 [16]. DPP9 also processes the mislocalized mitochondrial protein AK2 [18, 19] and mislocalized ER proteins harboring N-terminal DPP9 cleavage sites [29]. In all these cases, cleavage by DPP9 unmasks N-terminal degrons, thereby triggering the proteasomal degradation of these substrates through the N-degron patwhay [8]. Based on this mechanism, DPP9 regulates B-cell receptor signaling (via SYK), facilitates DNA damage repair (via BRCA2), and prevents the cytosolic accumulation of AK2 [16–19]. Several ubiquitin E3 ligases act sequentially of DPP9 in these pathways. These include CBL, members of the UBR family, and proteins of the inhibitor of apoptosis (IAP) family such as XIAP [17, 18, 29].

Also screens for DPP9 partners have been previously carried-out using classical approaches such as co-immunoprecipitations or yeast two-hybrid assays [17, 30–34]. These led to the identification of the scaffold protein Filamin A (FLNA) and the small ubiquitin-like protein modifier SUMO1, which regulate DPP9. Specifically, FLNA facilitates the interaction of DPP9 with its substrates SYK and BRCA2 [16, 17], while SUMO1 allosterically activates DPP9 [30]. A second group of interactors includes NLRP1, CARD8 and KEAP1, that are sequestered by DPP9, which interferes with their activity. KEAP1 is an adaptor of the BTB-CUL3-RBX1 E3 ubiquitin ligase. By interacting with KEAP1, DPP9 prevents the association of KEAP1 with its substrate NRF2, and leads to NRF2 stabilization and the modulation of oxidative stress responses [23, 32, 33, 35, 36]. Binding of DPP9 to NLRP1 and CARD8 prevents cleavage of Caspase-1 and inflammasome activation [21, 31, 34, 37–43]. While successful the classical screening approaches have some limitations. For example, co-immunoprecipitations and pull-down assays are not well suited for identification of dynamic and weak interactions, while yeast-two hybrid assays are performed outside the context of full-length proteins and their native subcellular localization.

To broaden our understanding of DPP9’s cellular functions, we set out an unbiased screen to map its interactome to identify proteins that are either sequestered by DPP9, or those that regulate this protease. For this, we utilized an optimized TurboID-based proximity labeling approach, which is exceptionally well suited for detecting dynamic interaction partners in living cells [44]. Along with established DPP9 interactors we identified and confirmed novel partners including DPP8, the ubiquitin E3 ligase CBL and deubiquitinating enzymes (DUBs) that preferentially target lysine-63–linked ubiquitin chains. Mechanistic studies with selected DUBs confirmed their interactions with DPP9 and uncovered a previously unrecognized crosstalk between DPP9 and components of the ubiquitin system as a context-dependent scaffold that modulates the assembly of protein complexes.

## Materials

### Reagents and tools table

**Table:** 

Experimental Models	Reference or Source	Identifier or Catalog Number
HEK293 FLP-In™ T-Rex™	Invitrogen™	R78007
HEK293 FLP-In™ T-Rex™ DPP9^KO^	[16]	
HEK293 FLP-In™ T-Rex™ DPP9^KO^ HA-miniTurbo-DPP9-S^WT^	This study	
HEK293 FLP-In™ T-Rex™ DPP9^KO^ HA-miniTurbo-DPP9-S^S730A^	This study	
HEK 293T	ATCC	CRL-1573
HeLa FLP-In™ T-Rex™	kind gift from Prof. Matthias Hentze	
HeLa FLP-In™ T-Rex™ DPP9^KO^	[45]	
Recombinant DNA	**Reference or Source**	**Identifier or Catalog Number**
Gateway™ pDONR™221 Vector	Invitrogen™	12536017
HaloTag empty vector	Promega	G6591
pcDNA3.1 (-)	Invitrogen	V795-20
pcDNA5^TM^FRT/TO	Invitrogen™	V601020
pCDNA5_FRT_TO_N-miniTURBO_GW	This study	
pcDNA5 FRT/TO HA-miniTurbo-DPP9-S^WT^	This study	
pcDNA5 FRT/TO HA-miniTurbo-DPP9-S^S730A^	This study	
pFC14K HaloTag^®^ CMV Flexi^®^ Vectors	Promega	G9661
pCDNA3_FLAG-DPP9	[30]	
pCDNA3_HA-DPP8	This study	
pFC14K_ABRO1-HaloTag	This study	
pFC14K_BRCC36-HaloTag	This study	
pFC14K_CYLD-HaloTag	This study	
pFC14K_SPATA2-HaloTag	This study	
pFC14K_DPP8-HaloTag	This study	
pFC32 NanoLuc^®^ Protein Fusion Flexi^®^ Vectors	Promega	N1341
pFC32K_ABRO1-NanoLuc	This study	
pFC32K_BRCC36-NanoLuc	This study	
pFC32K_CYLD-NanoLuc	This study	
pFC32K_SPATA2-NanoLuc	This study	
pFN21A HaloTag^®^ CMV Flexi^®^ Vector	Promega	G2821
pFN21A_HaloTag-ABRO1	This study	
pFN21A_HaloTag-BRCC36	This study	
pFN21A_HaloTag-CYLD	This study	
pFN21A_HaloTag-DPP9-S	This study	
pFN21A_HaloTag-SPATA2	This study	
pFN31 NanoLuc^®^ Protein Fusion Flexi^®^ Vector	Promega	N1321
pFN31K_NanoLuc-ABRO1	This study	
pFN31K_NanoLuc-BRCC36	This study	
pFN31K_NanoLuc-CYLD	This study	
pFN31K_NanoLuc-DPP9-S	This study	
pFN31K_NanoLuc-SPATA2	This study	
pOG44 Flp-Recombinase Expression Vector	Invitrogen™	V60052
Antibodies	**Reference or Source**	**Identifier or Catalog Number**
mouse anti-beta-actin	Sigma-Aldrich	AC-15
rabbit anti-CBL	Santa Cruz biotechnology, Inc	sc-170
rabbit anti-DPP9	abcam	ab42080 RRID: AB_731947
goat anti-DPP9	Self-made [11]	
Mouse anti-FLAG beads	Sigma-Aldrich	A2220
mouse anti-HA	BioLegend	901,501
rabbit anti-HA	Sigma-Aldrich	H6908
IRDye^®^ 680RD Streptavidin	LI-COR	926–68079
mouse anti-Tubulin	Santa Cruz	sc-32293
Secondary Antibodies	**Reference or Source**	**Identifier or Catalog Number**
IRDye^®^ 800CW donkey anti-Mouse IgG	LI-COR	926–32212
IRDye^®^ 680RD donkey anti-Mouse IgG	LI-COR	926–68072
IRDye^®^800CW donkey anti-Rabbit IgG	LI-COR	926–32213
IRDye^®^ 680RD donkey anti-Rabbit IgG	LI-COR	926–68073
donkey anti-goat Alexa-Fluor-488	Invitrogen	A-11055
donkey anti-rabbit Alexa-Fluor-594	Invitrogen	R37119
DuoLink in Situ PLA probe rabbit plus	Sigma-Aldrich	DUO92002
DuoLink in Situ PLA probe goat minus	Sigma-Aldrich	DUO92006
DuoLink in Situ Detection Reagents Red	Sigma-Aldrich	DUO92008
Oligonucleotides and other sequence-based reagents	**Reference or Source**	**Sequence**
ABRO1 + Sgf1 (nanoBRET)_fwd	Sigma - Aldrich	TAAGGCGATCGCCATGGCGGCGTCCATTTCGGGCTACA
ABRO1 + Sgf1 (nanoBRET)_rev	Sigma - Aldrich	TAGAGTTTAAACAATCTGGGAGGTCTGAGTGTTCCTGGGG
BRCC36 + Sgf1 (nanoBRET)_fwd	Sigma - Aldrich	ATAAGCGATCGCCATGGCGGTGCAGGTGGTGCAGGCG
BRCC36 + Sgf1 (nanoBRET)_rev	Sigma - Aldrich	GGCAGTTTAAACTTCTAGAGAAGAAAGTTCTTGCATAAGCTCTTCCTTTTCTTGTTGTAATTCCT
CYLD + Sgfl (nanoBRET)_fwd	Sigma - Aldrich	TAACGCGATCGCCATGAGTTCAGGCTTATGGAGCC
CYLD + Pmel (nanoBRET)_rev	Sigma - Aldrich	GTCCGTTTAAACTTTGTACAAACTCATTGTTGGACTCTG
DPP9-S WT + Sgfl (nanoBRET)_fwd	Sigma - Aldrich	GCGCGCGATCGCCATGGCCACCACCGGGACCCCAAC
DPP9-S WT + Pmel (nanoBRET)_rev	Sigma - Aldrich	GAAAGTTTAAACgAGGTATTCCTGTAGAAAGTGCAGCAACGTGACT
SPATA2 + Sgf1 (nanoBRET)_fwd	Sigma - Aldrich	CCTCGCGATCGCCATGGGGAAGCCCAGTTCAATGG
SPATA2 + Pmel (nanoBRET)_rev	Sigma - Aldrich	GAATGTTTAAACTCTGTACACGAGATGGGAGA
rev_attB2_DPP9	Sigma - Aldrich	GGGGACCACTTTGTACAAGAAAGCTGGGTcctaTCAgAGGTATTCCTGTAGAA
fw_attB1_DPP9S	Sigma - Aldrich	GGGGACAAGTTTGTACAAAAAAGCAGGCttCATGGCCACCACCGGGACCCC
Chemicals, Enzymes and other reagents	**Reference or Source**	**Identifier or Catalog Number**
Albumin (BSA) Fraction V (pH 7.0)	AppliChem	A1391
Alkaline Phosphatase (FastAP)	Thermo Scientific	EF0651
Benzonase	Sigma - Aldrich	E1014
Biotin	Sigma-Aldrich	B4369
Biozym LE Agarose	Biozym	840004
Blasticidin (solution)	InvivoGen	ant-bl-1
Carboxy Flexi Enzyme Blend	Promega	R1901
CytoPainter Phalloidin-iFluor 488 Reagent	Abcam	ab176753
DAPI (4’,6-Diamidin-2-phenylindol, Dihydrochlorid)	Invitrogen	D1306
Dimethyl sulfoxide (DMSO)	Sigma-Aldrich	D-2438
1,4 Dithiothreitol (DTT)	AppliChem	A1101
Doxycycline	Ratiopharm	PZN4314646
Fetal bovine Serum	PAN biotech	P30-3306
Fetal bovine serum, Tetracycline-free	PAN-Biotech	P30-3601
Flexi Enzyme Blend	Promega	R1852
Fluorescence mounting medium	Agilent Dako	S3023
Formaldehyde	Sigma-Aldrich	104,003
FuGENE HD Transfection Reagent	Promega	E2312
Gibco^®^ DMEM	ThermoFisher	41965-039
Gibco^®^ L-Glutamin	ThermoFisher	25030-024
Gibco^®^ 0,25% Trypsin EDTA	ThermoFisher	25200-056
HaloTag^®^ NanoBRET™ 618 Ligand	Promega	G9801
H-Gly‐Pro‐7‐amino‐4‐methylcoumarin (Gly-Pro-AMC)	Bachem	I-1225 0050
Hygromycin B	Gibco	10,687,010
Lipofectamine 3000	Invitrogen™	L3000001
NanoBRET Nano-Glo^®^ Detection Systems	Promega	N1662
Penicillin- Streptomycin	ThermoFisher	15140-122
Phalloidin-iFluor 488	Abcam	ab176753
Phosphate buffered saline	PAN biotech	P04-36500
Protein Assay Dye Reagent Concentrate	BioRad	500-0006
Protease inhibitor cocktail	Sigma - Aldrich	539,134
SafeView Classic	abm	G108
streptavidin magnetic beads	A2S	L00936
Software		
Biorender	https://BioRender.com	RRID: SCR_018361
Fiji (2012)	[46]	RRID: SCR_002285
GraphPad Prism	8.4.3	RRID: SCR_002798
Image Studio Lite	Version 5.2	LI-COR
NanoDrop 2000 Software	Thermo Fischer Scientific	RRID: SCR_018042
ShinyGO (2020)	0.82, [47]	RRID: SCR_019213
SnapGene	v5.3.1	
Perseus	v2.1.3	
UniProt	http://www.uniprot.org/	RRID: SCR_002380
Venny (2007–2015)	2.1	RRID: SCR_016561
Zen (blue edition)	version 2.3 and version 3.3	Zeiss

### Methods, experimental design and statistical rationale

#### Plasmid construction and recombinant DNA

The pcDNA5 FRT/TO miniTurbo backbone was generated by PCR cloning of the HA-miniTurbo sequence from Addgene plasmid #107,170 [44] into the pcDNA5 FRT/TO vector (Thermo Fisher Scientific, #V601020). Coding sequences for DPP9-S^WT^ and the catalytically inactive variant DPP9-S^S730A^ from pDONR221 DPP9-S^S730A^ and pDONR221 DPP9-S^WT^ were cloned into this backbone using the Gateway Cloning System (Thermo Fisher Scientific) following the manufacturer’s instructions. The resulting constructs were designated pcDNA5 FRT/TO HA-miniTurbo DPP9-S^WT^ and pcDNA5 FRT/TO HA-miniTurbo DPP9-S^S730A^. For NanoBRET assays, expression constructs were generated using the Flexi Vector System (Promega) according to the manufacturer’s protocol. All plasmids were sequence-verified and analyzed using SnapGene software. The following plasmids were used as templates: pcDNA3 HA-SPATA2, pcDNA3 TOPO CYLD V5 [48], pOZ-N-FH_KIAA0157 ABRO1/ABRAXAS2 (kind gift from Roger Greenberg Addgene plasmid # 27499 [49]), pDEST_LTR_N_FLAG-HA-BRCC3_IRES_puro (kind gift from Wade Harper Addgene plasmid # 22540 [50]).

#### Generation of stable cell lines (HEK293 DPP9 ^*KO*^ HA-mTB-DPP9-S^*WT*^, HEK293 DPP9^*KO*^ HA-mTB-DPP9-S^*S730A*^)

HEK293 DPP9^KO^ HA-mTB-DPP9-S^WT^ and HEK293 DPP9^KO^ HA-mTB-DPP9-S^S730A^ were generated using the Flp-In T-REx system (Invitrogen™). HEK293 DPP9 knockout (HEK293 DPP9^KO^) cells were established previously in the Flp-In T-REx background [16]. In short, HEK293 DPP9^KO^ cells were co-transfected with the Flp recombinase pOG44 and either pcDNA5 FRT/TO HA-miniTurbo-DPP9-S^WT^ or pcDNA5 FRT/TO HA-miniTurbo-DPP9-S^S730A^ using Lipofectamine 3000 (Invitrogen™) according to the manufacturer’s protocol. Following transfection, cells were selected using blasticidin and hygromycin B to ensure stable integration at the Flippase recognition target (FRT) site. Single colonies were isolated, expanded and screened for inducible expression of DPP9 upon doxycycline (Dox) treatment (1 µg/mL). Expression of the HA-mTB-DPP9 fusion proteins was validated by Western blotting following SDS-PAGE. Protein signals were visualized using the Odyssey CLx Imaging System (LI-COR).

#### Cell culture

All cell lines were maintained in Dulbecco’s Modified Eagle Medium (DMEM, ThermoFisher) supplemented with 10% fetal bovine serum, 2 mM L-glutamine, and 1% Penicillin/Streptomycin (100 U/mL Penicillin and 100 µg/mL Streptomycin). Cells were cultured at 37 °C in a humidified incubator and 5% CO_2_. For biotin labeling experiments, cells were grown in tetracycline-free FBS.

#### Indirect Immunofluorescence

HEK293 DPP9^KO^ HA-mTB-DPP9-S were seeded on poly-D-lysine coated glass coverslips (VWR) and incubated for 24 h at 37 °C and 5% CO_2_. Cells were fixed with 4% formaldehyde in phosphate buffered saline (PBS) for 10 min at room temperature, followed by permeabilization with 0.2% Triton X-100 in PBS for 5 min. After two PBS washes, cells were blocked with 2% bovine serum albumin (BSA) in PBS for 10 min. Primary antibodies were added for 90 min at 37 °C, followed by two PBS washes and incubation with the respective fluorescently labeled secondary antibodies for 45 min at room temperature. The cover-slides were washed with PBS and di-destilled H_2_O, then mounted in a fluorescent mounting medium (DAKO) with DAPI. Cell Imaging was performed using the Axiovert observer Z1 microscope with EC plan-Neofluar 63x/1.25 oil objective. Images were processed using Zen 2.3 (blue edition) and 3.3 pro blue edition (Zeiss) and figures generated with FIJI.

#### Proximity ligation assay

HeLa WT and DPP9^KO^ cells were seeded on coverslips at a density of 2 × 10^4^ cells/well and incubated for 24 h at 37 °C and 5% CO_2_. Fixation and permeabilization were performed as described above. Proximity Ligation Assay was carried out using the Duolink in Situ PLA Kit (Sigma-Aldrich) according to the manufacturer’s instructions. Cells were washed with PBS and blocked using Duolink Blocking Solution for 1 h at 37 °C, followed by incubation with primary antibodies against CBL and DPP9 for 1.5 h at 37 °C. Actin filaments were co-stained using CytoPainter Phalloidin–iFluor 488. Control coverslips were incubated with only one primary antibody to assess background PLA signal. After washing, cells were treated with PLA probes and detection reagents as per the manufacturer’s instructions. Coverslips were mounted in DAKO with DAPI fluorescent mounting medium and imaged using an LSM 880 confocal microscope, 63x NA 1.4 oil immersion objective (Zeiss). Images were processed using Zen black v. 2.3 (Zeiss) and analyzed using the Duolink ImageTool (Sigma).

#### DPP9 activity assay

Cells were lysed in ice-cold activity buffer containing 20 mM HEPES (pH 7.9), 1.5 mM MgCl₂, 10 mM KCl, 0.5% Triton X-100 and 1 mM DTT. Lysis was performed for 1 h at 4 °C, followed by centrifugation at 600 × g for 10 min at 4 °C. Protein concentration of the lysates was determined using a SPECTROstar Nano plate reader (BMG Labtech). For enzymatic assays, 14 µg lysates were incubated with 1 mM Gly-Pro-AMC in 384-well microplates. Fluorescence was measured at 380 nm excitation and 480 nm emission, EnSpire multimode plate reader (PerkinElmer). Fluorescence readings were taken every 60 s at 24 °C. Measurements were performed in technical triplicates. Data were analyzed, and graphs were generated using GraphPad Prism v8.4.3.

#### HA-mTB-DPP9 expression and biotin labeling

Cells were cultured in Dulbecco’s Modified Eagle Medium (DMEM) supplemented with 10% tetracycline-free FBS, 2 mM L-glutamine, 100 U/mL penicillin, and 100 µg/mL streptomycin. Dox (10 ng/mL) was added for the indicated time periods to induce expression of HA-mTB-DPP9. Biotin (50 µM) was added to initiate proximity labeling for the indicated durations (2–4 h).

#### Proximity labeling of miniTurboID-DPP9 under basal conditions and following thapsigargin treatment

For labeling of the DPP9 interactome under basal conditions, HEK293 DPP9^KO^ HA-mTB-DPP9-S^WT^ cells were seeded on 15 cm dishes and cultured in DMEM supplemented with 10% tetracycline-free FBS, as described above. Each condition was performed in two biological replicates, with two 15 cm dishes per replicate. Expression of HA-mTB-DPP9 was induced by the addition of Dox (10 ng/mL) for 4 h. HEK293 DPP9^KO^ cells treated with Dox served as control. After induction, the Dox-containing medium was removed, and biotin (50 µM) was added to initiate proximity labeling for the indicated durations (2, 3, or 4 h). For labeling in the presence of ER stress, HEK293 DPP9^KO^ HA-mTB-DPP9-S^S730A^ were plated and induced by Dox as described above. Following the removal of the Dox-containing medium, Thapsigargin (TG, 600 nM) was added together with biotin for 2 h. Control cells were similarly treated with TG and biotin, but did not receive Dox. Cells were harvested with cold PBS, centrifuged at 250 × g for 5 min at 4 °C. Cell pellets were snap-frozen in liquid nitrogen and stored at −80 °C, before lysis in 5 mL buffer containing 50 mM Tris-HCl (pH 7.5), 150 mM NaCl, 1 mM EDTA, 1 mM EGTA, 1% Triton X-100, 0.1% SDS, supplemented with protease inhibitor cocktail (1:1000; Sigma) and 250 U benzonase. Lysis was performed for 1 h at 4 °C with constant rotation, followed by centrifugation at 16,000 × g for 30 min at 4 °C. Supernatant were incubated with pre-washed streptavidin magnetic beads for 3 h at 4 °C with rotation. Beads were washed three times with RIPA buffer (without protease inhibitors), and twice with 50 mM ammonium bicarbonate.

#### On-bead protein digestion, peptide cleanup and quantification

Beads were transferred to fresh low-bind tubes and suspended in 200 µL of 50 mM ammonium bicarbonate (pH 8.0) containing 1 µg of sequencing-grade modified trypsin (Promega Cat# V5111). Digestion was carried out overnight at 37 °C followed by the addition of an additional 0.5 µg trypsin for a 2 h incubation at 37 °C. Peptides were recovered by transferring the supernatant to fresh tubes and washing the beads twice with 150 µL of 50 mM ammonium bicarbonate (ABC). All eluates, including the washes, were pooled. Samples were dried using a vacuum concentrator and resuspended in 20 µL of 0.1% formic acid (FA) in 2% acetonitrile (ACN). Peptides were desalted using C18 spin columns (PolyLC INC. Cat# TT200C1896) according to the manufacturer’s instructions. Eluted peptides were dried by SpeedVac and resuspended in 20 µL of 2% ACN with 1% FA.

#### LC-MS/MS analysis

Tryptic peptides were analyzed by LC-MS/MS using either a Q Exactive Plus mass spectrometer (Thermo Fisher Scientific) coupled to an Ultimate 3000 LC system (Thermo Fisher Scientific) via a nanospray ion source, or an Orbitrap Exploris 480 mass spectrometer (Thermo Fisher Scientific) coupled to an EvoSep One HPLC system (EvoSep Biosystems).

Q-Exactive Plus–Ultimate 3000 Analysis: Desalted peptides were separated by reverse-phase chromatography on a homemade analytical column (30 cm length, 75 μm inner diameter) packed with ReproSil-Pur C18-AQ resin (1.9 μm, Dr. Maisch GmbH, Germany). Chromatographic separation was performed using a 60-minute linear gradient from 5% to 28% ACN in 0.1% FA, followed by a 15-minute increase to 95% ACN, and a 10-minute wash at 95% ACN, at a flow rate of 0.15 µL/min. MS data were acquired in positive ion mode using data-dependent acquisition. Full MS scans were acquired over an m/z range of 300–1800 at a resolution of 120,000, with an AGC target of 3 × 10⁶ and a maximum injection time of 20 ms. The top 20 most intense precursor ions (charge states 2–7) were selected for higher-energy collisional dissociation (HCD) with a normalized collision energy (NCE) of 27. MS/MS scans were acquired at a resolution of 15,000, with an AGC target of 1 × 10⁵ and a maximum injection time of 30 ms. Dynamic exclusion was set to 20 s. Orbitrap Exploris 480–EvoSep One Analysis: Peptides were loaded onto EvoTips, washed twice with 20 µL of 0.1% formic acid, and kept moist with 150 µL of 0.1% FA until injection. Peptide separation was carried out on a 15 cm × 150 μm column packed with 1.9 μm C18 resin (EvoSep EV1106), using the manufacturer’s standard 88-minute method. Full MS scans were acquired from m/z 300 to 1500 at a resolution of 120,000, with an AGC target of 3 × 10⁶ and a maximum injection time of 25 ms. The top 20 most intense precursor ions were selected for HCD fragmentation and analyzed at a resolution of 17,500. Dynamic exclusion was set to 30 s.

#### Immunoprecipitation assays

Immunoprecipitation assays were carried out as described in [30]. In short, HEK293T cells were transfected with pcDNA3 HA-DPP8 and/or FLAG-DPP9. Cells were harvested 48 h later in ice-cold lysis buffer (2% HEPES (v/v), 110 mM KAc, 2 mM MgAc tetrahydrate, 1 mM EGTA) supplemented with 0.2% Tween 20. The homogenate was centrifuged at 100,000 g at 4 °C for 20 min. Supernatants were pre-cleared, followed by immunoprecipitation for 2 h at 4 °C with mouse anti-FLAG beads. After extensive washing with TB containing 0.2% Tween 20, bound proteins were eluted with FLAG peptide (0.5 mg/ml) in the same buffer.

#### NanoBRET assays

HEK293T cells were transiently transfected with NanoLuc- and HaloTag-fusion constructs using FuGENE HD (Promega) according to the manufacturer’s instructions. 24 h post-transfection, cells were treated with HaloTag NanoBRET 618 Ligand (Promega) or DMSO as a negative control, then seeded into white 96-well plates (Nunc™ MicroWell™, Thermo Scientific). Cells were incubated for 4–6 h at 37 °C in 5% CO₂ prior to measurement. For signal detection, NanoBRET Nano-Glo^®^ Substrate was added in Opti-MEM I Reduced Serum Medium (no phenol red), and luminescence was measured within 1,5 h using a SPARK Multimode Microplate Reader (TECAN). Donor emission was measured from 415 to 485 nm, and acceptor emission from 610 to 700 nm, at room temperature. For each protein tested, 2 to 4 tag configurations were evaluated in triplicate. The fusion protein pair yielding the highest corrected BRET signal was selected for subsequent donor saturation assays (DSAs).

For DSAs, HEK293T cells were transiently transfected with the optimized donor-acceptor plasmid pairs. The amount of acceptor plasmid (HaloTag-fusion vector) was titrated while the donor plasmid (NanoLuc-fusion) was held constant. Wells transfected with empty HaloTag vector or treated with DMSO served as negative controls. Raw BRET ratios were calculated as the ratio of acceptor signal (610–700 nm) to donor signal (415–485 nm). To correct for donor bleed-through, values from DMSO-treated control wells were subtracted from ligand-treated wells, yielding the corrected BRET ratio (miliBRET units (mBU)). Data were analyzed in Excel, and saturation binding curves were fitted using nonlinear regression (one-site specific binding model) in GraphPad Prism v8.4.3.

For disruption assays, the transiently transfected donor-acceptor ratio was fixed to the Kd value (half-maximal saturation) of the corresponding saturation binding curve from the DSA. To test for disruption, a titration of pcDNA3.1 HA-DPP9 plasmid was co-transfected with the fixed acceptor-to-donor ratio, while the total amount of plasmid was kept constant by varying the addition of pcDNA3.1(-) plasmid. Wells treated with DMSO served as controls. Measurement and calculations were done as described above.

#### Mass spectrometry data analysis

MS data analysis was conducted using FragPipe v.22.0 (https://fragpipe.nesvilab.org/) in DDA + mode [51] using the LFQ-MBR workflow with default parameters, except where noted. MSFragger v.4.2 was used for database searching against the *Homo sapiens* UniProt reference proteome (January 2024 release; 20,425 entries), supplemented with common contaminant sequences. The search enzyme was set to trypsin, allowing up to two missed cleavages. Carbamidomethylation of cysteine was set as a fixed modification, while methionine oxidation and protein N-terminal acetylation were included as variable modifications.

Peptide-spectrum matches (PSMs) were rescored using MSBooster and validated with Percolator, applying a false discovery rate (FDR) threshold of 1% at both the peptide and protein levels. Label-free quantification (LFQ) was performed using IonQuant. The match-between-runs option was disabled for the analysis under basal conditions, but enabled for the ER stress condition, with the “top runs” parameter set to 2. Protein intensities were computed using the MaxLFQ algorithm. Label-free quantification data were imported into Perseus v2.1.30 [52] where log₂ transformation and all statistical analyses were performed. Proteins were retained only if they had valid label-free quantification (LFQ) intensity values in all replicates of at least one experimental group. Missing values were imputed from a normal distribution (width = 0.3, downshift = 1.8). Differential abundance between groups was assessed using 1-sided-sample Student’s *t*-test. For the analysis under ER stress conditions, these results were further refined by selecting only proteins that were identified by at least three peptides in both miniTurbo-DPP9-S^S730A^ samples and either were absent in both control samples or showed at least eightfold higher spectral counts compared to the controls.

#### Statistics

ImageStudio Lite Version 5.2 software was used for the quantification of Western blots, which were visualized with LI-COR. For enzymatic activity assays, measurements from three technical replicates per data point are represented as mean and error ± SEM. Graphs were generated using the GraphPad Prism 8.4.3. software.

#### Bioinformatic analysis of proteomics data

Gene Ontology (GO) enrichment analysis was performed on proteins significantly enriched in DPP9-expressing samples relative to DPP9 knockout controls using the ShinyGO platform (version 0.80; [47]). The species was set to “human”, and enrichment was assessed across the Cellular Component, Molecular Function and Biological Process categories. Importantly, the list of all proteins identified in each individual proteomics experiment was used as the background reference set, rather than the full human proteome. This approach accounts for experiment-specific coverage and minimizes background bias in the enrichment results. Full STRING networks were generated for proteins significantly enriched in DPP9-expressing samples relative to DPP9 knockout controls using STRING database (v12.0, https://string-db.org/). The organism was set to Homo sapiens. Physical subnetworks with a high confidence score (0.7) were visualized. Clustering was performed using Markov Clustering (MCL) algorithm [53] with an inflation parameter of 3. Selected clusters of the full STRING were highlighted based on the Biological Process (Gene Ontology).

## Results

### Identification of DPP9-associated interacting partners by proximity biotin labeling

To expand our understanding of its biological functions, we aimed to to screen for the interactome of DPP9, and to identify novel binding partners, including those that bind only transiently to DPP9. For this we took an unbiased approach using the TurboID-based proximity biotin labeling. Importantly, biotin labeling occurs in living cells, thus enabling the detection of transient and context-specific interactions within their native subcellular environment. In this approach, a biotin ligase is fused to a protein of interest and labels lysine residues in proteins that are in close proximity (10–15 nm) [44], upon addition of biotin. To the best of our knowledge, only one other study applied proximity labeling to study proteases. This was done for proteases localized to the mitochondrial intermembrane space [54], a compartment with a highly restricted proteome of approximately 100–200 proteins. Our work represents the first use of this technique to map interactions of a broadly localized protease like DPP9.

**Fig. 1:**
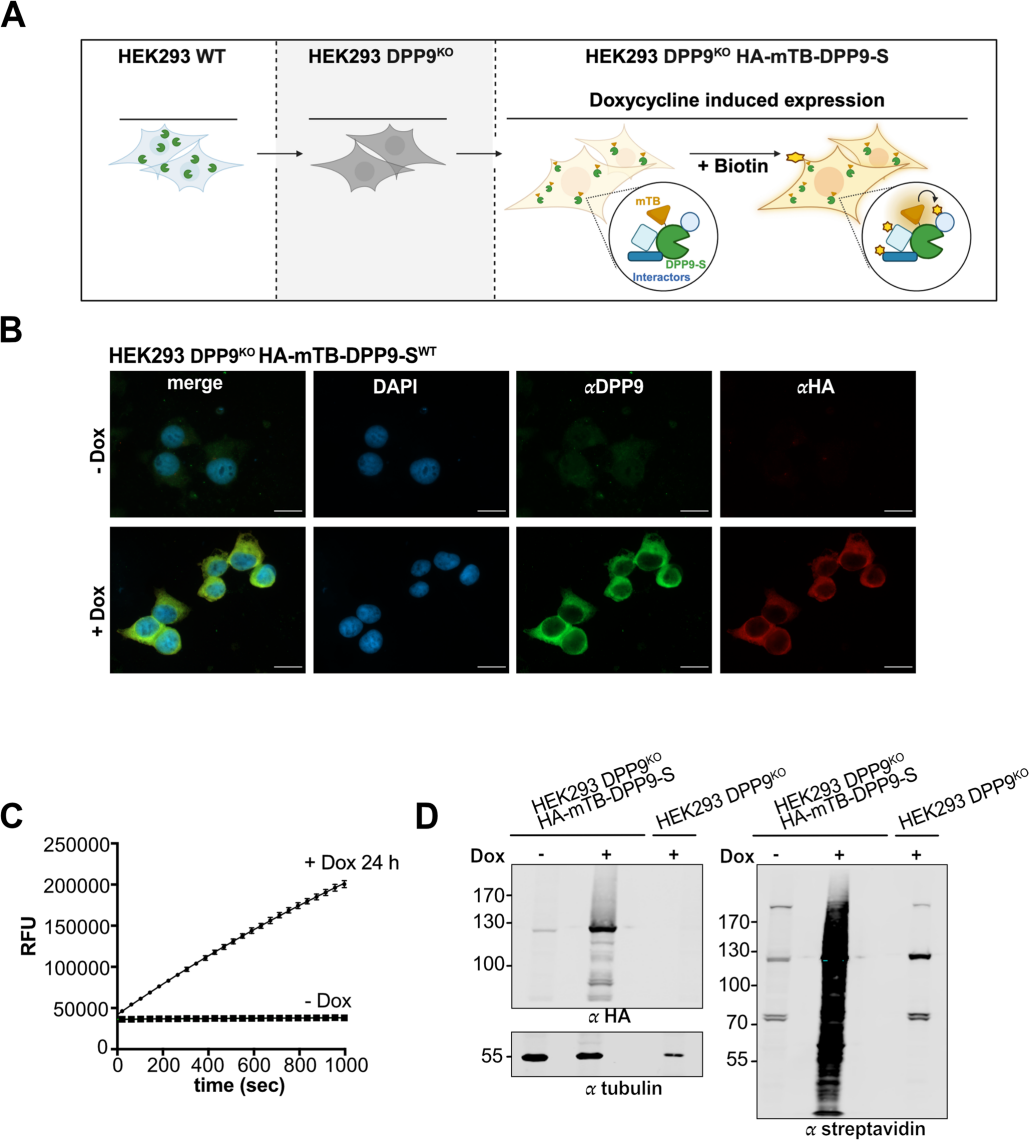
**Biotin labeling with HA-mTB-DPP9-S**^**WT**^**(A)** Schematic presentation showing the workflow used to generate the stable cell line HEK293 DPP9^KO^ HA-mTB-DPP9-S^WT^. Endogenous DPP9 was first CRISPRed out from HEK Flp-In^TM^T-REx^TM^−293 (HEK293) cells to generate HEK293 DPP9^KO^cells followed by stable integration of the HA-mTB-DPP9-S^WT^ construct. HA-mTB-DPP9-S^WT^ expression is induced by the addition of Dox. **(B)** Representative immunofluorescence images showing cytoplasmic localization of HA-mTB-DPP9-S^WT^ following Dox treatment. DAPI (blue) stains the nucleus; anti-DPP9 (green) and anti-HA (red) label HA-mTB-DPP9-S^WT^. Control cells were untreated. Scale bar 20 μm. **(C)** DPP9 activity assay using the artificial substrate Gly-Pro-AMC, and lysates from Dox treated and untreated cells. Hydrolysis of GP-AMC is measured as relative fluorescence units (RFU) over time. Data represent technical triplicates from a single experiment (*n* = 1), plotted as mean ± SEM. **(D)** Lysates of HEK293 DPP9^KO^ HA-mTB-DPP9-S^WT^ treated with Dox (200 ng/mL, 24 h) and biotin (50 µM, 2 h) show HA-tagged DPP9 expression and biotinylation of multiple proteins, as detected by fluorescently labeled streptavidin. Untreated (- Dox) and DPP9^KO^ were used as controls. Tubulin was used as a loading control

Briefly, DPP9-S^WT^ was fused to miniTurboID (HA-mTB), a smaller version of the biotin ligase (28 kDa) with enhanced activity (TurboID) [44]. Stable cell lines with a Dox inducible expression of HA-mTB-DPP9-S^WT^ in Flp-In™ T-REx^TM^−293 cells were generated, in which endogenous DPP9 was deleted (HEK293 DPP9^KO^ HA-mTB-DPP9-S^WT^) (Fig. 1A). Immunofluorescence imaging and Western blot analysis confirmed the Dox-induced expression and cytoplasmic localization of HA-mTB-DPP9-S^WT^. Lysates from Dox‑treated cells exhibited robust hydrolytic activity toward the artificial DPP substrate Gly‑Pro‑AMC, confirming that HA-mTB-DPP9-S^WT^ is enzymatically active and that the N-terminal mTB moiety does not interfere with the activity of DPP9 (Fig. 1B-D). For an initial screen of the DPP9 interactome, Dox was added for 24 h to induce the expression of HA-mTB-DPP9-S^WT^, followed by a 2 h incubation with biotin. Biotinylated proteins were captured on streptavidin beads, followed by on-bead digestion and mass spectrometry (MS) analysis. HEK293 DPP9^KO^ cells served as control (Fig. 1D).

**Fig. 2:**
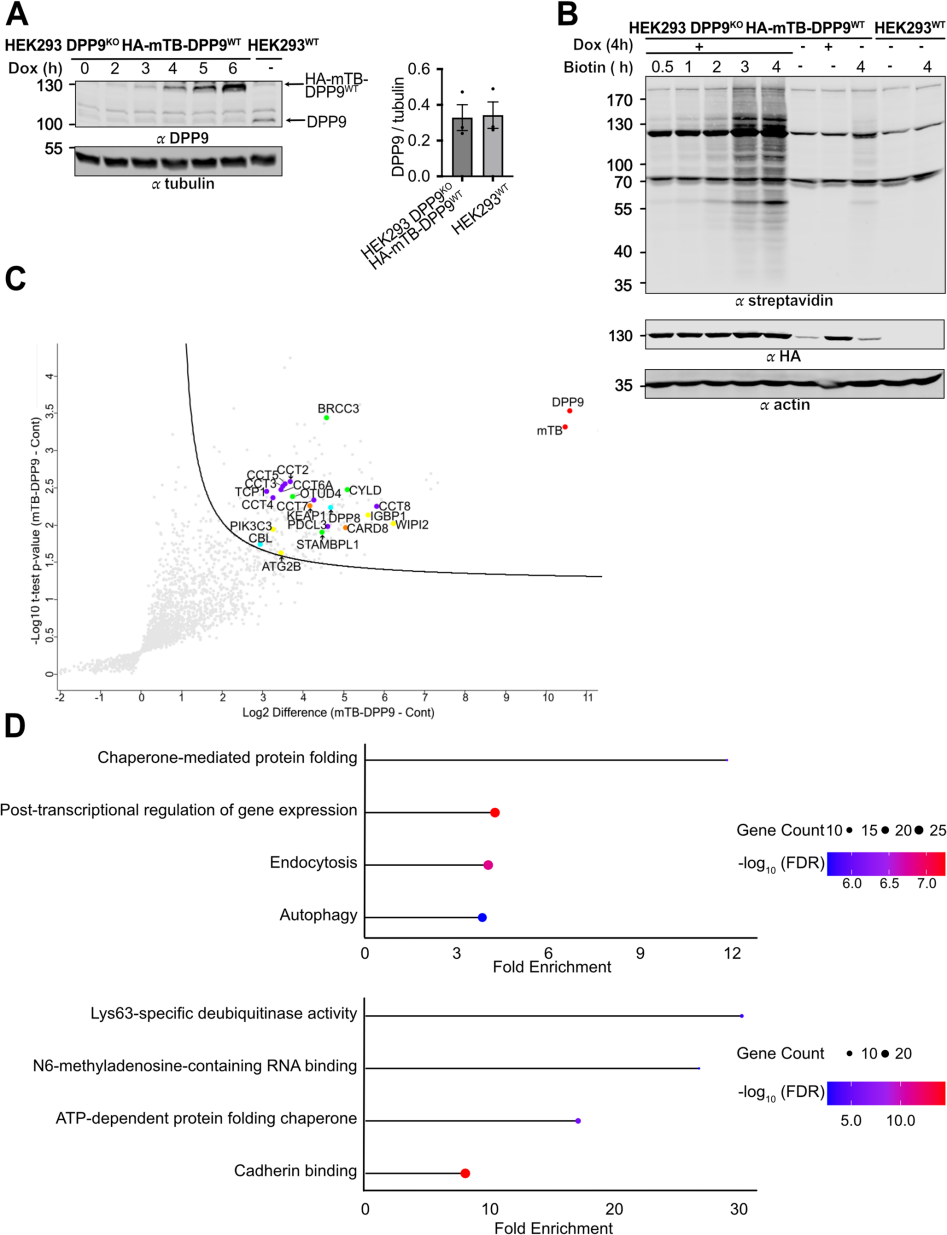
**Proximity labelling for HA-mTB-DPP9-S**^**WT**^ **and functional enrichment analysis**. **(A)** Representative Western blot showing that treatment with Dox (10 ng/mL, 4 h) induces HA-mTB-DPP9-S^WT^ expression to levels comparable to expression of endogenous DPP9 in HEK293^WT^ cells. Tubulin was used as loading control. The graph summarizes the results from three independent biological replicates showing the ratios of HA-mTB-DPP9^WT^/tubulin in HEK293 DPP9KO HA-mTB-DPP9^WT^ cells treated for 4 h with 10 ng/ml Dox, and the DPP9/tubulin ratio in HEK293^WT^ cells. **(B)** Time dependent biotinylation of proteins in HA-mTB-DPP9-S^WT^ expression, treated with Dox (10 ng/mL, 4 h). Dox-treated HEK293^WT^ was used as a negative control. Biotinylated proteins were detected by fluorescent streptavidin, β-actin was a loading control. **(C)** Volcano plot of biotinylated proteins identified by one-sided t-test comparing induced (HA-mTB-DPP9-S^WT^) and control samples from HEK293 DPP9^KO^. DPP9 and miniTurbo (mTB) are marked in red. Known DPP9 interactors, KEAP1 and CARD8, are highlighted in orange. Autophagy-related proteins are highlighted in yellow. K63-specific deubiquitinating enzymes are shown in green. ATP-dependent chaperones, including TRiC subunits are highlighted in purple. **(D)** Lollipop charts of the Gene Ontology term enrichment for biological processes and molecular functions. Dot size represents the number of genes associated with each term, and color indicates statistical significance (FDR-adjusted p-values, shown as -log₁₀)

To more closely reflect physiological conditions, the expression of HA-mTB-DPP9-S^WT^ was fine-tuned by titrating the Dox concentration and incubation times, achieving steady state levels at 4 h 10 ng/ml Dox treatment comparable to those of endogenous DPP9 in HEK293^WT^ cells (Fig. 2A). Different biotin labeling times showed robust labeling after 2 h of biotin exposure (Fig. 2B). Using these optimized conditions, a second proximity labeling screen was performed, with a shorter Dox-induction time, followed by biotin labeling in the absence of Dox. As a control, HEK293 DPP9^KO^ cells, were treated identically with Dox and biotin. Mass spectrometry analysis after on-bead digestion of the protein captured on streptavidin beads resulted in the identification and quantification of a total of 2251 proteins (Supplementary Table 1). To filter for high-confidence DPP9-associated candidates, a one-sided t-test was applied to identify proteins significantly enriched in HA-mTB-DPP9-S^WT^ samples compared to controls. Proteins were further filtered by requiring a minimum of two spectral count hits in all DPP9 samples and none in the DPP9^KO^ controls. This analysis yielded 234 promising candidate interactors. Among these, previously reported DPP9 binding partners such as CARD8 and KEAP1 were detected, supporting the validity of the approach. Analysis of this set revealed enrichment of autophagy-related proteins (e.g., WIPI2, IGBP1, PIK3C3, ATG2B), Lysine-63-specific deubiquitinating enzymes (e.g., CYLD, STAMBPL1, OTUD4, BRCC36), and ATP-dependent chaperones, including several T-complex protein 1 Ring (TRiC) Complex subunits (CCT4, TCP1, CCT7, CCT6A, CCT5, CCT8, CCT3, CCT2, and PDCL3) (Fig. 2C-D). Taken together, by titrating the cellular concentration of mTB-DPP9, the TurboID proximity labeling approach can be successfully applied to identify a short list of 234 putative DPP9 binding partners.

### DPP9 interacts with the E3 ligase CBL and with DPP8

**Fig. 3:**
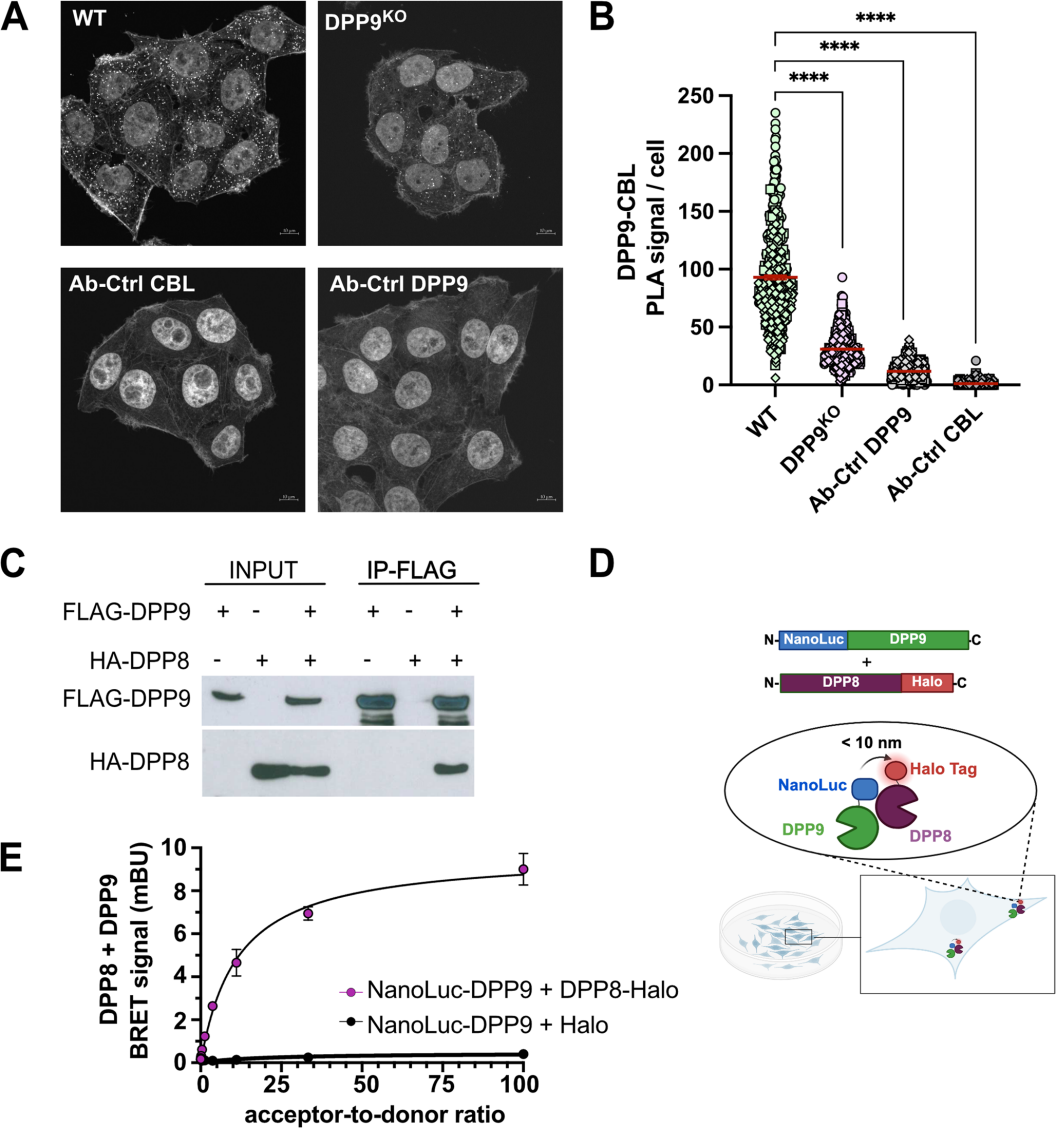
Validation for the interaction of DPP9 with CBL and DPP8.**(A)** PLA of HeLa^WT^ and DPP9^KO^ cells using antibodies against endogenous DPP9 and CBL. Representative images and **(B)** quantification of PLA events per cell (right) from three independent biological replicates (*n* = 3) are shown. WT cells display significantly more PLA signals than DPP9^KO^ and single antibody controls. Red bars represent mean ± SEM; ****p < 0.0001 by one-way ANOVA. (**C**) Co-immunoprecipitation assays showing that DPP8 interacts with DPP9. HEK293 cells were transfected with either HA-DPP8 or FLAG-tagged DPP9, or both together. Lysates were immunoprecipitated (IP) using anti-FLAG beads, and analyzed for both HA-DPP8 and FLAG-DPP9. (**D**) Schematic depiction of the NanoBRET assay. The NanoLuc Luciferase (NanoLuc) was N-terminally coupled to DPP9 and the HaloTag was C-terminally coupled to DPP8. (**E**) Donor Saturation Assay (DSA) for DPP9 with DPP8. Constant amounts of the donor fusion vector (DPP9) and increasing amounts of acceptor fusion vector (DPP8) were transfected into HEK293T cells. Negative controls included an empty HaloTag vector. DMSO controls accounted for donor-contributed bleed-through. The graph summarizes results from three biological replicate, performed in triplicates. Shown are mean and error ± SEM of specific binding. Graphs were generated using a one-site specific binding equation for fitting, assuming a single binding site

A closer inspection of the short list of putative DPP9 binding partners highlighted two proteins, the homolog DPP8 and the ubiquitin E3 ligase CBL, which is known to ubiquitinate multiple substrates, including the tyrosine kinase SYK, thereby targeting it for proteasomal degradation [55]. We previously demonstrated that DPP9 processes the N-terminus of SYK, to promote its proteasomal degradation, and that depletion of DPP9 reduces the interaction between SYK and CBL [17]. Given these findings indicating that both DPP9 and CBL contribute to SYK degradation, and that DPP9 promotes CBL-SYK interaction, we next investigated whether DPP9 and CBL form a complex within cells. To test this, proximity ligation assays (PLAs) were performed in HeLa^WT^ cells by targeting endogenous DPP9 and CBL. HeLa DPP9^KO^ cells were used as a negative control, along with the technical antibody controls. This approach demonstrated a close proximity between DPP9 and CBL in cells. The number of PLA events were significantly higher in wild-type cells compared to DPP9^KO^ controls, confirming a physical interaction between the two endogenous proteins in their native environment (Fig. 3A, B). Together, these results independently validate the proximity labeling data and provide the first direct evidence that DPP9 and endogenous CBL exist in close spatial proximity within cells.

DPP8, the closely related homolog of DPP9, is a less abundant member of the dipeptidyl peptidase family [10], (Fig. 2C). DPP8 shares an extensive sequence and structural similarity with DPP9, particularly within the catalytic domains. Both form stable homodimers that exhibit allosteric and cooperative substrate binding, displaying comparable substrate specificity and enzymatic kinetics in vitro [7, 10]. The enrichment of DPP8 in this screen prompted us to further investigate whether it interacts with DPP9. To explore this possibility, the crystal structure coordinates of DPP9 and DPP8 monomers [7] were employed for in silico modeling (Supplementary Fig. 1). Docking generated a single cluster of 200 complexes; the highest-ranking structure, corresponding to the most energetically favorable conformation was further used to define the DPP8-DPP9 interaction surface. This model predicts a stable heterodimer of DPP9-DPP8. Encouraged by these models, we performed co-immunoprecipitation assays in cells co-transfected with HA-DPP8 and FLAG-DPP9. Importantly, HA-DPP8 co-immunoprecipitated with FLAG-DPP9, indicating a high affinity interaction, whereas only background levels were observed in control reactions which did not include FLAG-DPP9 (Fig. 3C). To further examine this interaction in live cells, we performed NanoBRET assays, which monitor real-time protein interactions in live cells by detecting energy transfer between NanoLuc and HaloTag when within ~ 10 nm. Emission from NanoLuc, triggered by furimazine, provides a sensitive, quantitative measure of proximity (Fig. 3D). DPP8 was cloned in all combinations with either NanoLuc or HaloTag fused to the N- or C-terminus, to identify optimal tag placement. DPP9-S^WT^ was similarly fused to NanoLuc or HaloTag at the N-terminus consistent with the N-terminal position of the miniTurboID used for the proximity-labeling experiments (Fig. 1). The most effective tag combination pairs were selected based on signal intensity and the dynamic range, to generate donor saturation binding curves for NanoLuc-DPP9 and DPP8-HaloTag, demonstrating specific, saturable DPP8-DPP9 binding in live cells (Fig. 3E). Taken together, these data validate DPP8 as a novel binding partner of DPP9.

### Proximity biotin labeling during ER stress reveals an enrichment for proteins involved in RNA degradation

**Fig. 4:**
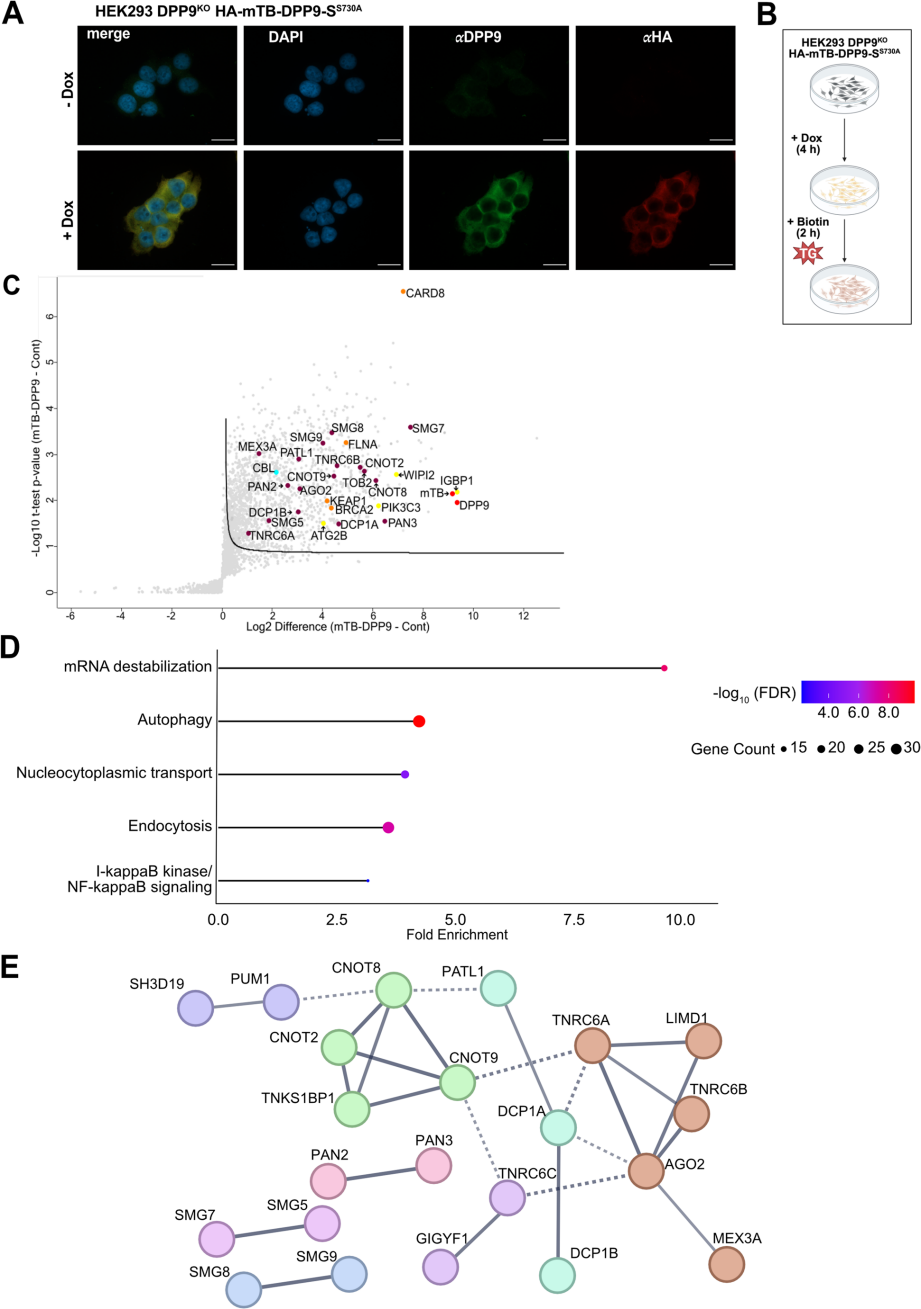
ER stress-dependent labeling by HA-mTB-DPP9-S^S730A^ reveals an enrichment for proteins involved in RNA degradation. **(A)** Representative immunofluorescence showing the Dox-induced expression of HA-mTB-DPP9-S^S730A^cells. DAPI (blue) stains the nucleus; anti-DPP9 (green), anti-HA (red). Scale bar 20 μm. **(B)** Workflow for biotinylation during ER stress. The expression of HA-mTB-DPP9-S^S730A^ was induced with Dox (10 ng/mL, 4 h). Following Dox removal, thapsigargin (TG) (600 nM) was added to induce ER stress, together with biotin (50 µM). **(C)** Volcano plot of biotinylated proteins identified by one-sided t-test comparing TG-treated versus control samples. **(D)** GO enrichment analysis for biological processes, based on proteins significantly enriched in the HA-mTB-DPP9-S^S730A^ samples. Highlighted categories include proteins associated with autophagy, RNA degradation, and endocytosis. Dot size represents the number of genes associated with each term, and color indicates statistical significance (FDR-adjusted p-values, shown as -log₁₀).** (E)** STRING interaction network showing a selected cluster of proteins enriched in HA-mTB-DPP9-S^S730A^ samples under ER stress. The displayed subnetwork includes functionally related proteins that are involved in RNA catabolic processes, mRNA destabilization, deadenylation-dependent Nuclear transcribed RNA catabolic processes and mRNA catabolic processes. This cluster was extracted from the broader STRING output to illustrate a coherent RNA surveillance module. Physical sub-network clusters are connected at a confidence score threshold of 0.7. Line thickness reflects the strength of evidence supporting each interaction. Such a cluster was not observed under basal conditions with HA-mTB-DPP9-S^WT^ (Supplementary table 1)

We generated a second stable cell line expressing HA-mTB-DPP9-S^S730A^, an enzymatically inactive variant of DPP9 in which the catalytic serine 730 is substituted with an alanine. This mutation allows substrate binding, prevents its cleavage and circumvents the substrate degradation by the N-degron pathway [16, 17]. The HA-mTB-DPP9-S^S730A^ cell line was used to expand the interactome screen and assess whether this assay can be applied to evaluate if the DPP9 landscape undergoes changes under stress conditions (Fig. 4A). To this end, we analyzed DPP9 interactome following a treatment with Thapsigargin (TG), a non-competitive inhibitor of the endoplasmic reticulum (ER) Ca^2^-ATPase. Consequently, TG treatment depletes calcium from the (ER) and thereby increases cytosolic calcium levels. The changes in calcium concentration lead, on the one hand, to proteostatic stress in the ER [56–58], and, on the other, inhibits late steps of autophagy [59, 60] that induces ER stress (Fig. 4B). Control cells were not treated with Dox, but were similarly exposed to TG and biotin. Mass spectrometry identified a total of 3,713 proteins across all conditions. Applying filters based on fold-change and spectral count thresholds yielded a final list of 325 candidate interactors of DPP9 (Fig. 4C, Supplementary Table 1). As in the previous screen, several known DPP9 interactors were recovered including KEAP1, CARD8 and FLNA further supporting the validity of the approach. The tumor suppressor BRCA2 was also identified, consistent with the hypothesis that catalytically inactive DPP9 can be used to trap substrate proteins (Fig. 4C). Notably, under these conditions, we also identified XIAP, an E3 ubiquitin ligase recently reported to mediate the proteasomal degradation of mitochondrial precursor proteins such as AK2, following DPP9-dependent exposure of an IAP-binding motif [18]. Interestingly, functional annotation of the 325 candidates revealed a marked enrichment of proteins involved in RNA degradation (Fig. 4C – E). A closer inspection using STRING interaction network analysis [61] revealed additional components of mRNA decay and surveillance pathways, including PAN2, DCP1B, SMG5, SMG7, SMG8, SMG9, MEX3A, AGO2, TNRC6A and TNRC6B, which were not as enriched in the absence of TG. These proteins form a densely connected subnetwork representing a coherent RNA surveillance module enriched under ER stress (Fig. 4E). These findings suggest that DPP9 may engage with RNA regulatory complexes, particularly in response to TG-induced ER stress, and highlight the benefits of using catalytically inactive DPP9 to uncover potential substrates and stress-responsive interactors.

### The interaction landscape of DPP9 includes Lys63-specific deubiquitinase complexes

**Fig. 5:**
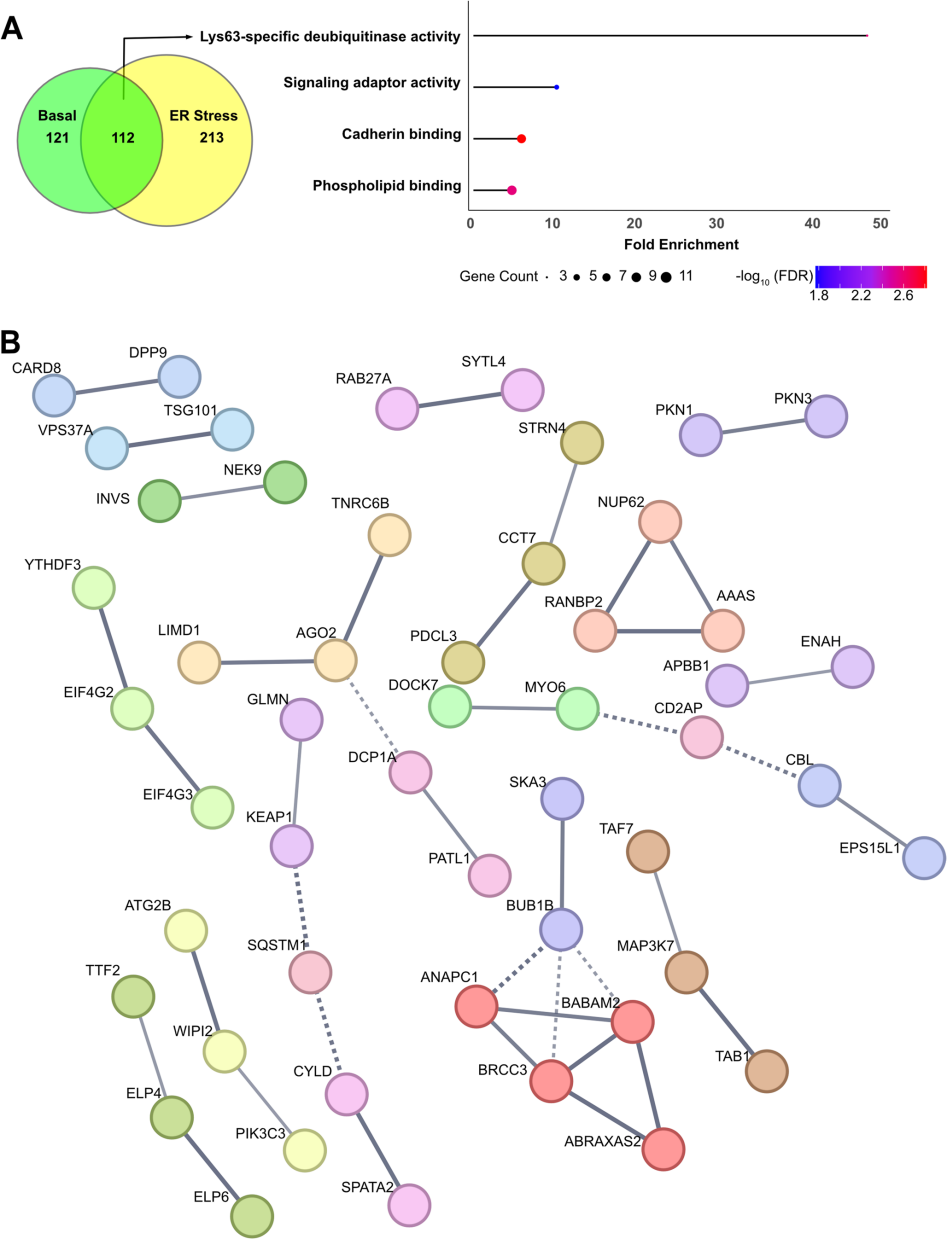
**Shared hits from dual DPP9 proximity labeling screens highlight Lys63-specific deubiquitinases and reveals functional protein clusters. (A)** Venn diagram showing the overlap of the two DPP9 proximity labeling experiments: one under basal conditions (green) and the other following ER stress induction (yellow). A total of 112 proteins were identified in both screens. Only proteins present in both screens were included in the GO enrichment analysis showing the highest fold enrichment for Lys63-specific deubiquitinase activity, alongside other molecular functions such as inositol phosphate binding and phosphatase binding. Dot size represents the number of genes associated with each term, and color indicates statistical significance (FDR-adjusted p-values, shown as -log₁₀). **(B)** STRING protein-protein interaction network of the 112 overlapping proteins, revealing tightly connected functional clusters at a confidence score threshold of 0.7. Line thickness reflects the strength of evidence supporting each interaction. Colored nodes represent query proteins and their first-shell interactors

A closer inspection of both screens highlighted a subset of 112 proteins significantly enriched in both DPP9 proximity labeling experiments - one conducted under basal conditions (Fig. 2) and the other during TG-induced ER stress (Fig. 5A and Supplementary Table 3). GO analysis of the molecular function terms among this subset of proteins revealed a strong enrichment for Lysine 63-specific deubiquitinating activity including canonical K63 DUBs such as CYLD and BRCC36 (also known as BRCA1/BRCA2-containing complex subunit 3) (Fig. 5A). To gain further insight into the functional relationships among those proteins enriched in both screens, we constructed protein-protein interaction networks using the STRING database. Clustering analysis identified 22 distinct clusters at a confidence score of 0.7. One of those clusters included BRCC36/BRCC3 and ABRO1/ABRAXAS2, BRE/BABM2, along with ANAPC1, BUB1B and SKA1 (Fig. 5B). BRCC36 is the catalytic subunit of two distinct multiprotein DUB complexes: the BRISC complex and the BRCA1-A complex [62–64]. The catalytic core of both complexes consists of the same three subunits: BRCC36, BRE and MERIT40/BABM1, but differ in their regulatory subunits and physiological functions. The BRISC complex includes the scaffold protein ABRO1 and can bind the associated metabolic enzyme SHMT2, whereas the BRCA1-A complex contains the scaffold protein ABRAXAS1 along with RAP80, and BRCA1. These compositional differences underline their distinct biological roles: BRISC complex is best characterized for its function in immune signaling, where it removes K63-linked ubiquitin chains from key substrates such as NLRP3 and IFNAR1, promoting inflammasome activation and enhancing type I interferon signaling, respectively [62, 64–66]. In contrast, the BRCA1-A complex is primarily involved in DNA double-strand break repair by homologous recombination [67, 68]. In line with their distinct functions, BRISC is predominantly localized to the cytoplasm, though it can also be detected in the nucleus, while BRCA1-A is nuclear and is recruited to DNA damage foci [62]. Importantly, in our proximity labeling experiments we identified not only BRCC36, but also BRE, a component of the core subunit, as well as ABRO1 of the BRISC complex (Fig. 5B). We did not identify ABRAXAS1, and although RAP80 was detected, its levels did not differ from the control, indicating that these two BRCA1-A–specific subunits were not enriched as direct DPP9-S interactors. In addition to BRCC36, we also identified CYLD, another K63-specific DUB. CYLD is recruited to signaling complexes by the Spermatogenesis-associated protein 2 (SPATA2) which acts as a scaffold to bridge CYLD to proteins such as HOIP/LUBAC at the TNF-receptor signaling complex [69–72]. This interaction is critical for the negative regulation of NF-κB signaling and inflammasome control.

### DPP9 hinders the interaction between CYLD and SPATA2

**Fig. 6:**
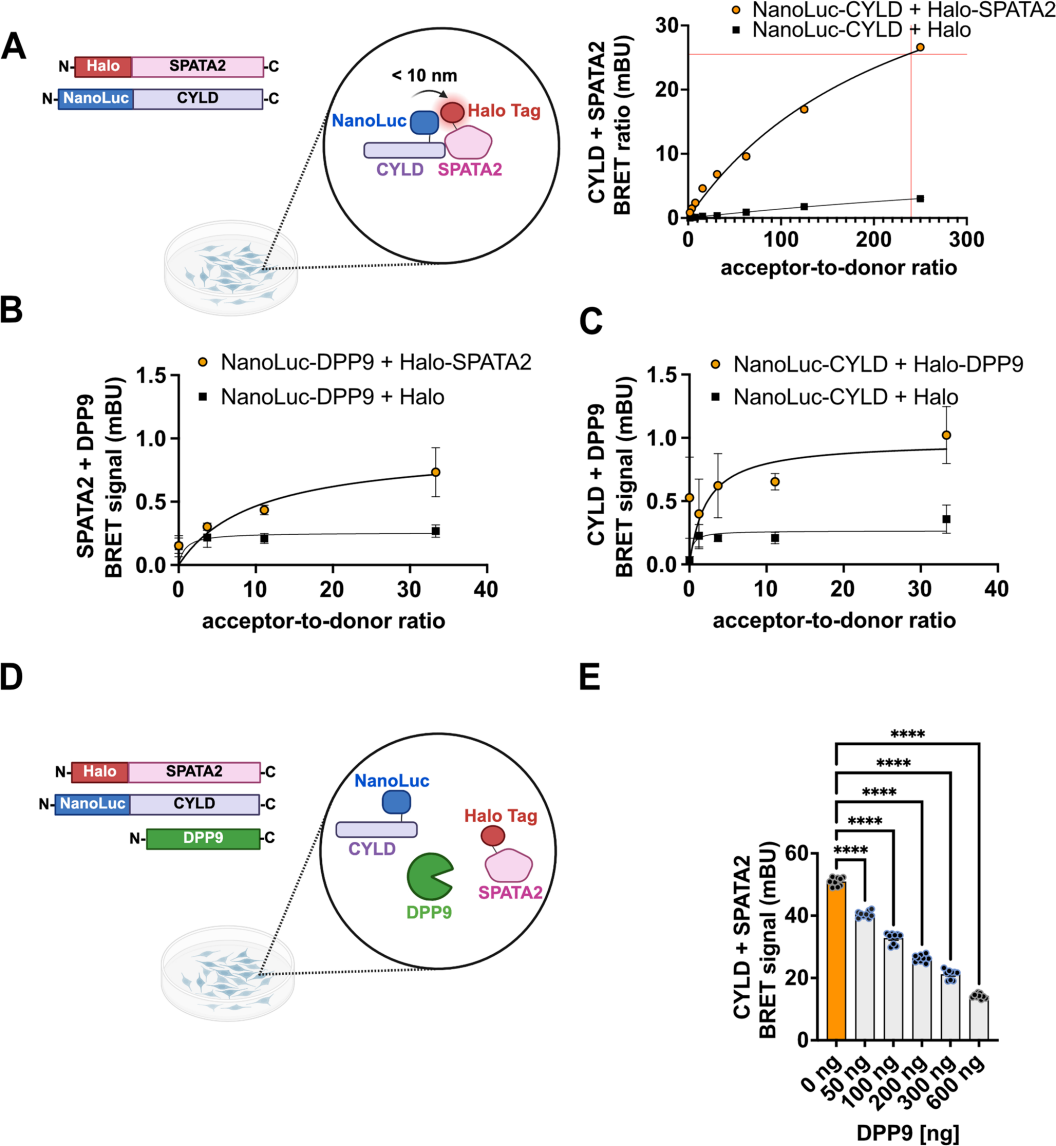
DPP9 competes out the CYLD and SPATA2 interaction in live cells.**(A)** Schematic depiction of the NanoBRET assay constructs. For DSA of CYLD with SPATA2, constant amounts of donor fusion vectors and increasing amounts of acceptor fusion vectors were transfected into HEK293T cells. Negative controls included an empty HaloTag vector. DMSO controls accounted for donor-contributed bleed-through. The graph summarizes results from one biological replicate, performed in triplicates. Shown are mean and error ± SEM of specific binding. Graphs were generated using a one-site specific binding equation for fitting, assuming a single binding site. **(B**,** C)** DSA for DPP9 with SPATA2 (B) or with CYLD (C) were performed as described in (**A**). Shown are results from three biological replicates, each performed with three technical replicates (*n* = 3). **(D)** Schematic depiction of the setup of NanoBRET assay to test the effect of DPP9 on the CYLD-SPATA2 interaction. **(E)** NanoBRET assays showing that increasing concentrations of DPP9 result in fewer interactions between SPATA2 and CYLD. Nano-BRET assays were performed with NanoLuc-CYLD and HaloTag-SPATA2 at an acceptor-to-donor ratio of 240:1, in the presence of increasing concentration of HA-DPP9-S^WT^ or empty vector for control reactions. Shown are results from 3 independent biological replicates, each performed with 3 technical replicates. Mean and error ± SEM of specific binding are illustrated, analyzed by a one-way Anova (p**** >0.0001)

Given the identification of both CYLD-SPATA2 and BRCC36-containing complexes in our proximity labeling screens, we next sought to determine whether DPP9 influences these interactions in cells. Using NanoBRET assays, we first examined CYLD and SPATA2 interactions. The most effective tag combination pairs were selected to generate saturation binding curves for NanoLuc-CYLD and HaloTag-SPATA2, confirming that the NanoBRET system could accurately detect their interaction in live cells (Fig. 6A). We then used this approach to test the interaction of DPP9 with each component. Similarly, the best combination pairs were identified, followed by saturation binding curves for NanoLuc‑DPP9 with HaloTag‑SPATA2, and for NanoLuc‑CYLD with HaloTag‑DPP9. These demonstrated specific, saturable binding (Fig. 6B, C), confirming the proximity‑labeling results and validating CYLD and SPATA2 as novel DPP9 partners.

To test whether DPP9 affects this interaction, HEK293 cells were co-transfected with NanoLuc-CYLD and HaloTag-SPATA2 at the defined ratio, along with increasing amounts of DPP9 (Fig. 6D). Control samples were transfected with an empty vector in place of DPP9. Strikingly, DPP9 co-expression resulted in a significant and concentration-dependent decrease in the NanoBRET signal between CYLD and SPATA2 (Fig. 6E), indicating that DPP9 interferes with their association. Taken together, these results confirm that DPP9 interacts with both CYLD and with SPATA2, and show that DPP9 negatively regulates their proximity.

### DPP9 regulates the formation of the BRISC complex

**Fig. 7:**
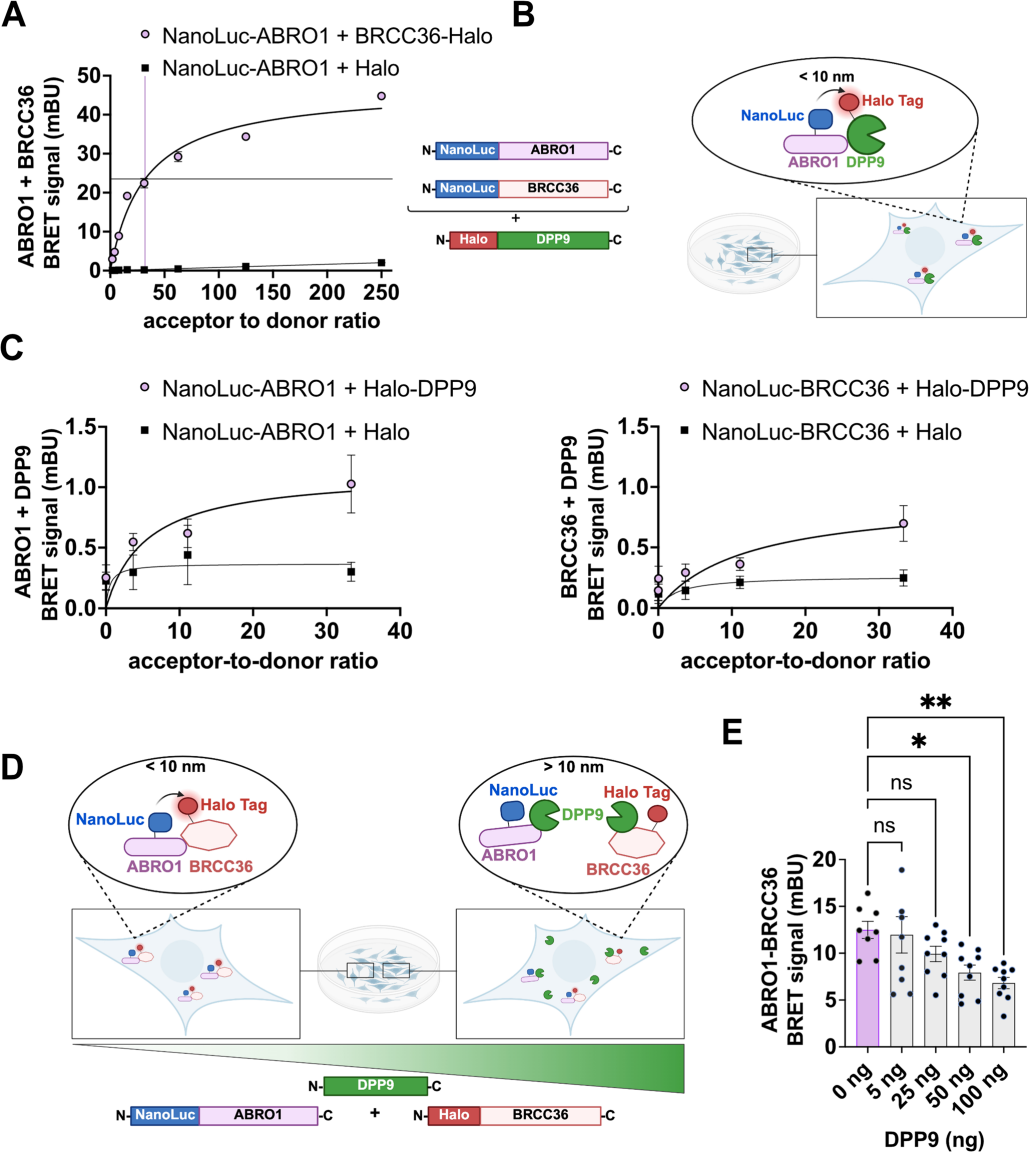
DPP9 limits the binding of BRCC36 and ABRO1 in live cells.**(A)** Saturation binding curves demonstrate a specific interaction of BRCC36-HaloTag to NanoLuc-ABRO1 in HEK293T. Negative controls included an empty HaloTag vector and DMSO-treated cells. BRET signal is plotted against the acceptor-to-donor ratio. Data are shown as mean ± SEM from one biological replicate with three technical replicates. Curves were fitted using a one-site specific binding model. **(B)** Schematic depiction of the NanoBRET assay constructs applied for the NanoBRET assays in (**C**). The NanoLuc Luciferase was N-terminally coupled to ABRO1 or BRCC36, and the HaloTag was coupled N-terminally to DPP9. **(C)** Donor saturation curves showing interaction for NanoLuc-ABRO1 (left) or NanoLuc-BRCC36 (right) with HaloTag-DPP9, demonstrate specific saturable interaction in HEK293 cells. Data represent mean ± SEM from three independent biological replicates, each with three technical replicates. Curve fitting was performed using a one-site binding model. **(D)** Schematic of BRET assay design showing the proximity-dependent interaction between NanoLuc-ABRO1 and BRCC36-HaloTag, modulated by DPP9. When DPP9 is not present, ABRO1 and BRCC36 are in close proximity (< 10 nm), allowing energy transfer. In the presence of DPP9, this interaction is disrupted (> 10 nm), reducing BRET signal. **(E)** Increasing levels of DPP9 reduce the NanoBRET signal between ABRO1 and BRCC36. Cells were transfected with NanoLuc-ABRO1 and BRCC36-HaloTag at a fixed acceptor-to-donor ratio of 32:1, along with increasing amounts of HA-DPP9-S^WT^ or control vector. Data are presented as mean ± SEM from three biological replicates, each with three technical replicates. Statistical analysis was performed using one-way ANOVA (*p = 0.027; **p = 0.0046; ns, not significant)

**Fig. 8:**
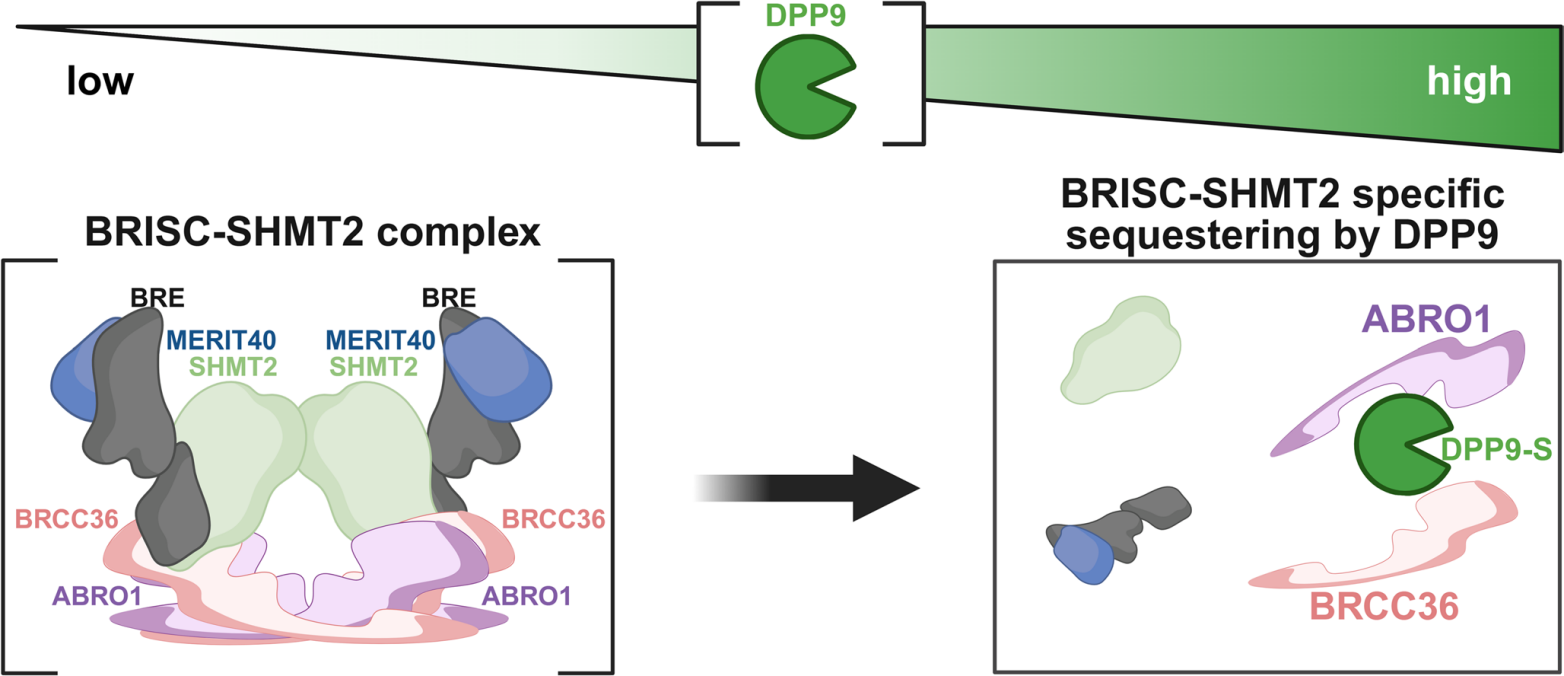
**Proposed model for DPP9-mediated modulation of BRISC and BRCA1-A complex assembly.** Under normal conditions BRCC36 assembles with ABRO1, BRE, MERIT40 (and SHMT2) to form the BRISC-SMHT2 complex. We propose that DPP9-S (cytosolic DPP9 isoform) binding to BRCC36 and ABRO1 disrupts these interactions either by preventing BRISC complex formation or destabilization of the existing complex

Next, we asked whether DPP9 similarly influences the association between BRCC36 and ABRO1, two key components of the BRISC complex. Cryogenic Electron Microscopy structures of the BRISC complex (PDB id:6H3C [62]), have shown that it forms a dimeric assembly of the three core subunits, BRCC36, BRE and MERIT40, which associates with two copies of ABRO1 and two copies of the inhibitory protein SHMT2 [73]. Within this architecture, BRCC36 and ABRO1 are positioned in close proximity and with direct interaction. To assess this interaction using NanoBRET, we tested multiple tag configurations and identified NanoLuc-Abro1 and BRCC36-HaloTag as the most effective donor-acceptor pair. DSA using this combination showed a specific and saturable interaction with half-maximal binding observed at an acceptor-to-donor (BRCC36-to-ABRO1) ratio of 32:1 (Fig. 7A). Importantly, NanoBRET DSA demonstrated that DPP9 interacts with both ABRO1 and BRCC36 in live cells, confirming the results of the BioID (Fig. 7B, C). We then tested whether DPP9 modulates their interaction by co-transfecting HEK293 cells with NanoLuc-ABRO1, BRCC36-HaloTag at the defined donor-acceptor ratio, along with increasing amounts of DPP9-S^WT^, or an empty control plasmid (Fig. 7D). Notably, DPP9 expression led to a concentration-dependent reduction in the NanoBRET signal between ABRO1 and BRCC36 (Fig. 7E). Together, these results demonstrate that, similarly to its effect on the CYLD–SPATA2 complex, DPP9 negatively regulates the assembly or stability of the BRISC complex by reducing the proximity between BRCC36 and ABRO1. Figure 8.

## Discussion

DPP9 plays a role in a wide range of physiological processes, and alterations in its expression have been associated with cancer development and immune-related disorders [8, 9]. For a comprehensive understanding of DPP9’s cellular functions and regulatory mechanisms we sought to define its molecular landscape using proximity labeling with the miniTurboID, an approach well suited for capturing both stable, and importantly, transient interactions within live cells. To minimize non-specific labeling and improve specificity, we generated stable cell lines expressing HA-mTB-DPP9 under Dox control, titrated its expression to approximate endogenous DPP9 levels, and maintained a short biotin labeling time. While background labeling was still present, these optimizations improved signal quality, and in combination with statistical filtering and comparative analysis, enabled the identification of a short list (*n* = 234 for basal conditions, and *n* = 325 for ER stress) of DPP9-associated proteins. These included known interactors such as KEAP1, CARD8, and FLNA, thereby validating the approach for mapping the interactome of DPP9 [17, 32, 34]. As with other high-throughput interaction assays, the dataset may include false-positives, direct and indirect interactors, thus additional validation is required to confirm selected candidates.

### DPP9’s connection to autophagy and mRNA decay proteins

Several proteins involved in autophagy initiation and membrane remodeling, including WIPI2, ATG2B, and PIK3C3 were highly enriched both under basal conditions and also in the presence of thapsigargin (see more below). These factors contribute to phagophore formation and autophagosome nucleation, suggesting that DPP9 may participate in the early stages of the autophagy pathway. The detection of IGBP1, a regulator of PP2A signaling linked to autophagy control [74], further supports this possibility. These associations complement a recent report implicating DPP9 in autophagy regulation in breast cancer cells and highlight the need for further investigation into this mechanistic role [25].

Expanding the analysis of the DPP9 interactome across different cell types, different physiological or stress conditions will be important for delineating its context-dependent molecular networks and understanding whether and how DPP9-mediated interactions adapt to cellular stress. Here, we employed Thapsigargin, which induces proteostatic stress in the ER [56, 58], and inhibits late steps of autophagy [59, 60]. Under these conditions, we observed an enrichment for proteins involved in mRNA decay and translational repression. These included components of the CCR4–NOT complex (CNOT2, CNOT8 and CNOT9) and the PAN2–PAN3 complex, both of which contribute to deadenylation, the first and rate-limiting step in mRNA decay. We also identified SMG5, SMG7, SMG8 and SMG9, components of the nonsense-mediated mRNA decay pathway along with AGO2, a core component of the RNA-induced silencing complex (RISC), which has been implicated in targeting ER-localized transcripts for degradation during stress adaptation [75]. Recent work on *C. elegans* reported a functional link between the DPP9 homolog (DPF-3) and AGO2 [76] although no interaction was detected using immunoprecipitation. Our proximity labeling data suggest that this association may be transient or stress-dependent. Together, these observations point to a potential role for DPP9 in modulating mRNA turnover and silencing in response to proteotoxic stress. They are also consistent with the growing evidence that mRNA decay pathways, beyond the canonical IRE1-dependent decay (RIDD), contribute to transcriptome remodeling during ER stress [75, 77–82]. Future studies will focus on validating these interactions and determining the functional consequences of DPP9 binding to mRNA decay machinery, autophagy initiation, and DPP8.

### DPP9 - DPP8 complexes

Here we identified DPP8 as a novel DPP9 partner. DPP8 was of particular interest, since it shares a high homology and similar structure with DPP9, and is similarly localized to the nucleus and the cytosol. Several studies have suggested that DPP8 has some overlapping functions with DPP9, for example in pyroptosis, where DPP9 however appears to have the dominant role [37, 38]. Beyond this, DPP8 and DPP9 also exhibit distinct biological functions. To name a few, DPP9, but not DPP8 plays a role in maturation of antigens for presentation on MHC class I molecules, promotes DNA repair and is critical for neonatal survival [8, 10, 83–85]. Our modeling highly supports the hypothesis that DPP8 and DPP9 form a heterodimer which shares a similar dimerization surface as the two homodimers. Likewise, the immunoprecipitation data and the NanoBRET assays all point to a stable interaction between the two proteins. Whether indeed they form a heterodimer, or a heterotetramer of homodimers will need to be further investigated but raises the intriguing possibility that these proteases form a functional DPP8-DPP9 complex, expanding current understanding of their potential substrate repertoire, cooperative or differential roles in cellular regulation.

### DPP9 interacts with ubiquitin E3 ligases

Our experimental approach revealed that DPP9 associates with several E3 ubiquitin ligases, most notably XIAP, CBL and the adaptor protein KEAP1. This is particularly interesting given DPP9’s known function in N-terminal processing, which can expose N-terminal degrons and thereby mark the substrates for ubiquitination and proteasomal degradation [8, 16, 17, 19, 29]. Although previous studies have demonstrated functional cooperation between DPP9 and several E3 ligases (UBRs, IAPs and CBL) for targeting proteins to degradation, direct proximity and potential physical association of these ligases with DPP9 has not been reported so far. XIAP mediates the degradation of DPP9-cleaved AK2 [18] and its identification as a DPP9 interactor under ER stress conditions (Fig. 4C) supports a cooperative role of XIAP and DPP9 in regulating proteostasis. Importantly, our data provide physical support for interaction with DPP9. CBL in particular, plays a well-characterized role in the turnover of the tyrosine kinase SYK [86]. We previously showed that DPP9 and CBL both regulate the stability of SYK, more specifically that DPP9-mediated cleavage of SYK’s N-terminus is a prerequisite for CBL-dependent ubiquitination and degradation [17]. Here, we show that the two proteins DPP9 and CBL are in close proximity in cells (Fig. 3). Additionally, we also recovered FLNA in this screen, an actin-binding scaffold protein required for DPP9-SYK interaction [17] and known to support CBL-mediated ubiquitination of EGF Receptor [87]. These observations raise the possibility that DPP9, FLNA, and CBL make up a multi-protein functional complex that facilitates recognition and turnover of SYK and potentially other substrates. Formation of such complexes which include DPP9 and an E3 ligase, are expected to minimizes off-target effects, and allow for tighter spatial and temporal control of degradation, making the pathway more precise and responsive to cellular signals.

### DPP9 interferes with K63-linked deubiquitinating complexes

In addition to E3 ligases, we identified several deubiquitinating enzymes as potential DPP9-associated proteins. These include STAMBPL1, OTUD4, CYLD, and BRCC36, all of which are known to cleave K63-linked polyubiquitin chains, which are involved in non-proteolytic signaling pathways. Among those, we validated the interactions of DPP9 with CYLD, and with its binding partner SPATA2, as well as with BRCC36 and its partner ABRO1, all known to participate in immune signaling. Importantly, DPP9 expression led to a decrease in NanoBRET signals between SPATA2-CYLD and BRCC36-ABRO1 pairs, showing that DPP9 negatively regulates these complexes. On the molecular level, one possible explanation for the reduced signal is that DPP9 trims the N-terminus of SPATA2, CYLD, BRCC36 or ABRO1 followed by proteasome degradation, as was seen for example for Syk, BRCA2 and AK2. BRCC36 and ABRO1 both display possible DPP9 cleavage sites at their N-terminus, however, as all four proteins were N-terminally tagged, we consider it less likely that the observed reduction in signal is a result from substrate cleavage by DPP9. An alternative model suggested by the data is that DPP9 physically interferes with complex formation or alters the spatial arrangement of the interacting proteins, consistent with a non-proteolytic regulatory role. In this model DPP9 contributes to regulating the composition and function of DUB complexes by modulating the spatial organization of protein assemblies (Fig. 8).

CYLD predominantly deconjugates K63- but also M1-linked polyubiquitin chains from specific substrates, thereby regulating NF-κB signaling, and facilitating cell death in response to TNF stimulation. The adaptor SPATA2 mediates the recruitment of CYLD to the linear ubiquitin assembly complex (LUBAC), particularly at signaling platforms such as the TNF receptor complex, enhancing CYLD activity [88]. Our data showing that DPP9 reduces the formation of the CYLD–SPATA2 axis, suggest that DPP9 may modulate NF-κB driven inflammatory responses. This may be particularly relevant to the NLRP3 inflammasome, where CYLD was reported to facilitate its assembly and activation [89]. DPP9 has previously been shown to suppress inflammasome activity by sequestering NLRP1 and CARD8 [31, 39, 42, 90]. Our findings raise the question whether DPP9 influences pyroptosis also via deubiquitination of NLRP3 complex components through CYLD. Collectively, these results position DPP9 as a broader modulator of inflammasome signaling, acting both through direct interactions with inflammasome sensors and via upstream control of ubiquitin signaling.

Similarly, we show that DPP9 outcompetes BRCC36 from binding to ABRO1, suggesting that DPP9 may interfere with BRISC complex formation or stability possibly by sterically hindering key protein-protein interactions. The BRISC complex regulates diverse processes including mitotic spindle assembly, immune signaling, and hematopoiesis, by targeting among others Aurora B kinase, IFNAR1, and JAK2 [65, 91, 92]. DPP9-mediated disruption of the BRISC formation may lead to different cell-dependent outputs, including defects in interferon signaling.

Moreover, since BRCC36 is a catalytic subunit shared by both of the BRISC and BRCA1-A complex [93], it is possible that DPP9 may similarly hinder the BRCC36-ABRAXAS1 interaction in the BRCA1-A complex. In our screens we identified DPP9 interactions with ABRO1, a BRISC-specific scaffold protein, but not with ABRAXAS1 or RAP80 which define the BRCA1-A complex. This may not be surprising taking into account that the BRCA1-A complex is nuclear, whereas the screen was conducted with DPP9-S which resides in the cytosol, and not with the nuclear variant DPP9-L. Additionally, our experiments were conducted under basal and ER stress conditions, rather than under conditions of DNA damage. It is therefore possible that DPP9 interacts with the BRCA1-A complex, only in response to DNA damage. Previous work has shown that the two complexes exist in fine balance in vivo and that reduction of ABRO1 results in an increase of the interaction of BRCC36 with RAP80 and ABRAXAS1 of the BRCA1-A complex [64]. Therefore, an alternative scenario is that DPP9 promotes the transition from BRISC to BRCA1 complex by destabilizing BRCC36-ABRO1 interaction, thereby facilitating the interaction of BRCC36 with ABRAXAS1. Whether DPP9 does regulate the BRCA1-A complex, similar to the BRISC, requires further investigation, with potential links to DNA repair. In a broader context, it is also worth considering whether DUBs themselves might regulate DPP9. While we found no evidence in the current literature for DUBs acting on DPP9 directly, such regulatory feedback is conceptually plausible.

In summary, our proximity-based proteomic screens provided a robust and systematic strategy to map DPP9-associated proteins under basal and stress conditions. Our data expand the known DPP9 interaction network, and suggest that DPP9 acts as a context-dependent scaffold that modulating mRNA decay, autophagy initiation and the assembly and activity of ubiquitin-associated signaling complexes under basal and stress conditions. Given this multifunctionality, therapeutic strategies targeting DPP9 should account not only for its catalytic activity but also for its scaffolding functions, which may have distinct and context-specific impacts on cellular homeostasis and immune regulation.

## Supplementary Information

Below is the link to the electronic supplementary material.

Supplementary Material 1 (DOCX 2.36 MB)

Supplementary Material 2 (XLSX 2.90 MB)

## Data Availability

The mass spectrometry proteomics data have been deposited to the ProteomeXchange Consortium via the PRIDE [94] partner repository with the dataset identifier PXD067345. (Reviewer access: Username: reviewer_pxd067345@ebi.ac.uk, Password: bxGDRwB7VaZD).

## Abbreviations

ABRO1/ABRAXAS2: BRISC complex subunit Abraxas 2
ABC: Ammonium bicarbonate
ACN: Acetonitrile
AGO2: Protein argonaute-2
BRCA1: Breast cancer type 1 susceptibility protein
BRCA2: Breast cancer type 2 susceptibility protein
DAPI: 4′,6-diamidin-2-phenylindol, dihydrochloride
DMSO: Dimethylsulfoxide
Dox: Doxycycline
DPP8: Dipeptidyl peptidase 8
DPP9: Dipeptidyl peptidase 9
DSA: Donor saturation assay
DUB: Deubiquitinating enzyme
ER: Endoplasmic reticulum
FA: Formic acid
FBS: Fetal bovine serum
FLNA: Filamin A
Gly-Pro-AMC: Gly‑Pro‑7‑amido‑4‑methylcoumarin hydrobromide
GO: Gene ontology
HA-mTB: HA-tagged miniTurboID biotin ligase
HCD: Higher-energy collisional dissociation
HEK293: Human embryonic kidney 293 cells
IAP: Inhibitor of apoptosis
KO: Knockout
LFQ: Label-free quantification
mBU: MiliBRET units
mTB: MiniTurboID biotin ligase
MS: Mass spectrometry
NanoBRET: NanoLuciferase Bioluminescence Resonance Energy Transfer
PBS: Phosphate buffered saline
PLA: Proximity ligation assay
RFU: Relative fluorescence units
SPATA2: Spermatogenesis-associated protein 2
STRING: Search Tool for the Retrieval of Interacting Genes/Proteins
SUMO1: Small ubiquitin like protein modifier 1
TG: Thapsigargin
