# Supplementary material for: Proximity labeling reveals non-catalytic interactions between DPP9 and ubiquitin signaling complexes
Source: Cell Mol Life Sci. 2026 Feb 4;83(1):93. doi: 10.1007/s00018-025-06021-z (PMC12876531; doi:10.1007/s00018-025-06021-z)
Supplement: Supplementary file 1 — Supplementary Material 1 (DOCX 2.36 MB) [file 18_2025_6021_MOESM1_ESM.docx]

Homodimeric structures of DPP9 (green, PDB ID: 6eor) and DPP8 (blue, PDB ID: 6eop) with a defined interaction surface of residues in distance of < 4 Å to corresponding molecule (left). Illustration of an *in-silico* molecular docking approach (HADDOCK2.4) to predict the structure of a possible DPP8/9 Heterodimer (right). The interaction surface between the two homodimers and the heterodimer is highlighted as transparent surface.


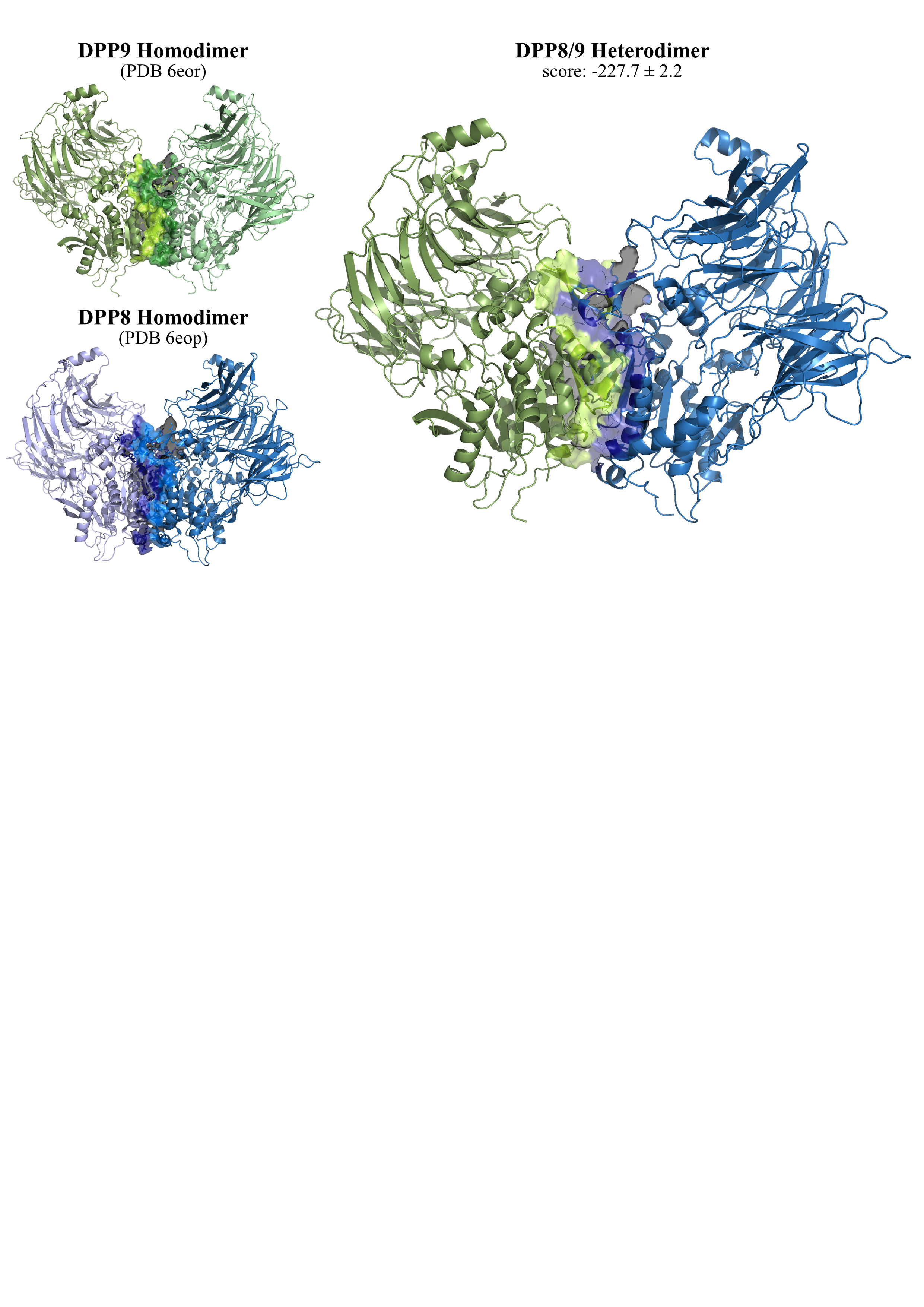


**In silico molecular docking:**

Shown are the solved crystal structures of DPP8 (PDB ID: 6eop) and DPP9 (PDB ID: 6eor) [1] . These structures were further used to identify the respective residues that are part of the interaction surface of each homodimer. As such, residues of each molecule within 4 Å to the 2^nd^ molecule were designated using PyMOL. Under the specified conditions, the interaction surface of the respective homodimers is formed by 43 residues of each DPP9 monomer and by 44 residues of each DPP8 molecule.

For *in silico* prediction of a possible heterodimer, the molecular docking software HADDOCK2.4 was used [2, 3] . For docking, the coordinates of a DPP8 and a DPP9 monomer were entered as molecules. Predefined residues of the homodimeric surface were set as active residues. This docking approach generated a single cluster of 200 complexes. The best ranked structure (according to the energetically most favorable conformation) was further used to define the interaction surface between DPP8 and DPP9 (residues within 4 Å to the 2^nd^ molecule) again. Noteworthy, the amount of residues involved in the interaction surface of the heterodimer decreased only slightly for DPP9 (40) and DPP8 (41) in comparison to the homodimers. The figure was made with Pymol Molecular Graphics System version 3.1.6.1.

1. Ross B, Krapp S, Augustin M, et al (2018) Structures and mechanism of dipeptidyl peptidases 8 and 9, important players in cellular homeostasis and cancer. Proc Natl Acad Sci 115:E1437–E1445. <https://doi.org/10.1073/pnas.1717565115>

2. Honorato RV, Koukos PI, Jiménez-García B, et al (2021) Structural Biology in the Clouds: The WeNMR-EOSC Ecosystem. Front Mol Biosci 8:729513. <https://doi.org/10.3389/fmolb.2021.729513>

3. Honorato RV, Trellet ME, Jiménez-García B, et al (2024) The HADDOCK2.4 web server for integrative modeling of biomolecular complexes. Nat Protoc 19:3219–3241. <https://doi.org/10.1038/s41596-024-01011-0>
